# Multi-scale attentional feature fusion-based traffic sign detection using image dehazing process of SPP-CAE and improved optimization of YOLOv9 model

**DOI:** 10.3389/frai.2026.1903277

**Published:** 2026-08-26

**Authors:** Thiyagarajan V, Vidhyapathi CM

**Affiliations:** School of Electronics Engineering, Vellore Institute of Technology, Vellore, Tamil Nadu, India

**Keywords:** Adaptive YOLOv9, feature pyramid network, multi-scale attentional feature fusion, SPP-CAE, traffic sign detection

## Abstract

For Intelligent Transportation Systems (ITS), traffic sign detection plays an essential role for autonomous vehicles to validate the condition of the roads more precisely. However, the environmental concerns, such as obstacles, lighting, and weather, may have an impact on the performance of the existing traffic sign detection systems. To overcome these problems, a model that uses a multi-scale-based learning network and image dehazing approach is developed in this work for traffic sign detection. At first, the required images are collected, and the input images undergo the pre-processing step. In this stage, the images utilize the Spatial Pyramid Pooling with Convolutional Autoencoder (SPP-CAE) for performing the image dehazing since the image is affected by unfavorable weather conditions. Finally, the pre-processed images are subjected as input to the object detection model, which employs the Multiscale Attentional Feature Fusion-based Feature Pyramid Network, incorporating Adaptive YOLOv9 (MAFF-FPN-AYv9). Here, the Multiscale Attentional Feature Fusion-based Feature Pyramid Network (MAFF-FPN) helped to extract the pertinent features and processed them under the YOLOv9 model for detecting the objects. For further improvement, the parameters present in the YOLOv9 model are optimally tuned by using the Stochastic Apiary Organizational Optimization with Population Amendment Strategy (SAOO-PAS). Lastly, the performance of the model is validated and examined by various metrics. With accuracies of 96% and 97% on the BDD100K and TT100K datasets, respectively, the results show how well this system performs, proving that the suggested model achieves superior results in detecting the traffic signs.

## 1 Introduction

Roadway marking and traffic signs play a crucial role in guaranteeing effective traffic flow and road safety (Chen and Luo, 2024). Notifying the drivers regarding the road conditions and their problems is one of the main objectives. To make accurate decisions based on the speed limitation, safety improvement, and driving protection, detecting the traffic signs has become an essential one for autonomous vehicles (Khalid et al., 2023). It also helps in increasing the effectiveness of the traffic detection process by preserving the safety in Intelligent Transportation Systems (ITS) (Trappey and Shen, 2024). Thus, the ITS helps in managing the traffic and improving road usage while reducing traffic congestion through the accurate traffic data analysis. Additionally, traffic sign detection is essential for autonomous vehicles to work efficiently, guaranteeing appropriate speed limits, road direction, and route optimization. As a result, travel time and fuel consumption are reduced, and in due course, improved path planning is streamlined (Han et al., 2024). However, various environmental factors, including light quality, reflection, and backdrop occlusion from trees, buildings, and cars, have an impact on the traffic sign detection process. In comparison to traffic sign detection, these elements are suggested to achieve greater efficiency (Ji et al., 2024).

The main problems with roads nowadays are injuries and other vehicle-related problems, such as clogged roads, low construction capacity, and poor road maintenance. The main problems with the existing traffic sign recognition techniques are a complex workplace, difficult mobile deployment, and inaccurate detection of small objects (Ren et al., 2024). The extra units will increase the storage cost of the detector and decrease its speed detection capability for in-car installation. Road sign sensors operate with traffic signs that have uniform designs but a variety of features, in contrast to common challenges such as habitat variability and significant feature diversity (Soylu and Soylu, 2023). Effective traffic sign feature extraction is difficult for normal object recognition systems, which often and unintentionally capture background features while avoiding particular traffic signal information (Gezgin and Alkan, 2024).

Importance of real-time datasets in autonomous driving scenarios: In autonomous driving environments, the traffic sign identification models need to work on real-time data sources. These data sources capture the dynamic road conditions, such as occlusions, motion blur, critical weather conditions, dynamic traffic flow, and changing illumination. Unlike the static image data sources, the real-time driving data sources offer various traffic scenarios, which resemble the practical conditions. Hence, implementing the identification techniques capable of executing real-time data with low latency and high accuracy is highly important. Thus, these models can guarantee secure vehicle navigation, timely decision making, and better perception. The publicly available data sources, like BDD100K and TT100K, include large-scale traffic scenarios gathered under various environmental conditions. It makes them suitable data sources for estimating the real-time applicability and efficiency of the traffic sign identification techniques.

### 1.1 Motivation

There have been some problems in designing effective and robust detection models to provide accurate road safety despite the significant advancements (Lu et al., 2024). Also, these challenges may lead to unpredictability and complexity in learning the original condition of the driving patterns, which may have a high impact on the accuracy and efficiency of the models. The environmental changes have become one of the main problems. The obscured and distorted traffic sign can hinder the vision of the drivers, which may sometimes lead to death (Shen et al., 2023). The aspects, such as drizzle, fog, sleet, and sun glare, make the detection models more problematic to identify the indicators consistently, even under poor circumstances. The different types of traffic signs pose another problem (Luo et al., 2024). Various regional areas and countries can have diverse road signage, which may result in significant variations in the shapes, colours, and symbols used. Moreover, another one of the challenges is the occlusion (Kamal et al., 2020). Sometimes, the traffic signs may be blocked fully or partially by some of the objects, including billboards, street lights, and trees, etc. Finally, the computational efficiency is the foremost problem, which may hinder the real-time applicability of the model by creating another level of complexity. In addition, continuously, the autonomous driving devices obtain the image streams from the onboard cameras. These systems demand identification algorithms to process real-time data with low latency under dynamic environmental scenarios. Hence, estimating the detection techniques employing large-scale data sources is significant for illustrating their practical applicability. Fog, rain, snow, or haze can impair vision, obscure images, and cause signs to be overlooked or detected incorrectly. Additionally, trees, cars, or other roadside items may occasionally and often completely or partially block traffic signs, making detection even more difficult. In addition to these environmental concerns, traffic signs are often small, particularly on high-speed roads or in cities where some signs may be grouped (Lin et al., 2019). This increases the difficulty of reliable detection, especially in real-time applications where prompt decisions are required (Tian et al., 2019). Lastly, it can be challenging to recognize signs that are fading, damaged, or covered in dirt, which makes it difficult to ensure that all pertinent signs are detected regardless of their state (Yuan et al., 2017). To solve all these issues, there is a necessity to develop an effective model that has the ability to balance the accuracy and speed in detection. Thus, this work develops an enhanced model to handle the occlusion, varying lighting patterns, and diversity of the traffic signs. By leveraging the ability of deep learning, the proposed system can provide safety and smooth navigation for autonomous vehicles.

### 1.2 Novelty

The novelty of the designed work is not only in the combination of various methods but also in the utilization of effective data processing methods across image enhancement, optimization, and multi-scale feature learning under degraded visibility scenarios. The main hypothesis of the designed work is that traffic sign detection performance under critical weather conditions can be highly enhanced when visibility restoration, multi-scale feature extraction, and parameter tuning are jointly performed within a single learning architecture. Most importantly, the utilization of the SPP-CAE-aided dehazing is performed as a feature restoration method that improves the downstream feature distinction for low-contrast and small traffic signs. This enhanced signal quality directly improves the MAFF-FPN block. This block is developed to collect the contextual data across different spatial regions. In addition, instead of employing constant YOLOv9 parameters, the designed SAOO-PAS optimization algorithm presents an effective parameter tuning strategy that improves the reliability of the detection process. Thus, these models demonstrate great improvement in the traffic sign identification under adverse environmental scenarios.

### 1.3 Contributions

The significant contributions of the developed model are as follows.

- A unified traffic sign detection approach is implemented by combining image restoration, multi-scale feature learning, and optimization for effective identification under critical weather conditions. The model is estimated on large-scale data sources, such as BDD100K and TT100K. It illustrates the designed model’s suitability in real-time autonomous driving environments.
- An SPP-CAE-aided image dehazing approach is suggested for preprocessing and feature improvement. It enhances the separability of low-contrast and weather-affected traffic sign features, thus improving the detection performance.
- The MAFF-FPN-AYv9 is designed by integrating MAFF-FPN with YOLOv9 for detecting traffic sign patterns. This method also improves the contextual feature representation across distinct spatial regions, thus enhancing the robustness.
- The SAOO-PAS is implemented to optimize the parameters of the YOLOv9 technique by presenting an adaptive strategy, which results in enhanced accuracy and detection stability.

### 1.4 Organization

This section elucidates the paper’s organization. Section 2 gives the literature review; an overview of the developed traffic sign detection model is given in Section 3; image pre-processing for dehazing is described in Section 4; a proposed mechanism for object detection is given in Section 5; results are discussed in Section 6; and the conclusion is given in Section 7.

## 2 Literature review

Liu et al. (2023) have suggested a UCN-YOLOv5 model that was developed based on the UNet Convolutional Network YOLOv5 architecture. This network suggested a new backbone by integrating UNet with the convolution network to improve the feature extraction ability. The improved system performed well on the CCTSDB2021 and LISA roadway sign databases. The accuracy of road sign identification has greatly increased over the baseline YOLOV5 models. Wang T. et al. (2024) have developed an improved multi-scale traffic sign identification method, MMW-YOLOv5, based on the YOLOv5 technique. The Multi-Scale Fusion Network (MSFNet) could identify even the small objects more accurately by using its enhanced feature extraction capability. The results proved that the precision of this model was 87.1% using the TT100K dataset.

Vehicle-Mounted Adaptive Traffic Sign Detector (VATSD) was developed by Wang et al. (2024b) in 2024, and this model was effective in detecting both small and larger signs under varying conditions of the traffic. Three diverse datasets were employed to analyze the performance of this model by using the adaptive filtering model for enhancing the image quality. The precision of this model was 7.6% higher than that of some other existing methods on the same datasets. Rani et al. (2024) have proposed an effective model, named Haze Removal based on Traffic Sign Detection and Recognition (DLHR-TSDR). The TSDR component in the proposed DLHR-TSDR was used to highlight the roadway marking more precisely by receiving the data from the previous component. By using the CURE-TSD dataset, the proposed DLHR-TSDR attained 99.01% accuracy, surpassing the traditional approaches. Zhang L. et al. (2025) have proposed Traffic Sign Detection utilizing Detection Transformer (TSD-DETR). The multi-scale attention element presented in this model improved the capacity to detect the complicated and small signs of the traffic. The ablation study revealed that the feature extraction module used here was more effective in providing accurate detection results.

Kuppusamy et al. (2023) have proposed YOLOv7 and Convolutional Block Attention Module (CBAM), which was applied to identify the signs of the roadway traffic. This research evaluated the variation of using a tiny dataset by managing the greatest accuracy score using configurations for adaptive optimizers. The high degree of accuracy showed how well the suggested model worked for the traffic sign identification assignment. In 2024, Zhao et al. (2024) have developed the Traffic Sign Detection-YOLO (TSD-YOLO), a unique system that improved the precision and resilience of road sign identification by integrating Mamba and YOLO innovations. This work cleared the path for the next developments in technology for autonomy, in addition to strengthening road sign recognition and tracking in these devices. Wang et al. (2024a) have implemented a portable, multi-scale roadway sign analyzer that could be mounted on an automobile. First, the attention absorption approach was used to propose an improved feature pyramidal structure. Further, multi-dimensional attention maps were integrated with extracted features, and then allocated with weights based on the significance of the data. The appropriate detection head was developed on the object’s scale to increase the precision of identifying the target. The experiment’s findings demonstrated that the suggested approach not only had an outstanding detection precision of 50.3% but was also comparable with different cutting-edge techniques.

Juneja et al. (2023b) have presented a hybrid Convolutional Neural Network (CNN) for performing image dehazing, and this model was processed using the coarse transmission map. The wavelet and inverted wavelet components were integrated to develop the hybrid CNN model, which finally generated an estimated transmission map. The experimental values obtained from the proposed work have been validated with the conventional works to ensure the generated images with the existing dehazing approaches. Juneja et al. (2023a) have developed an Aethra-Net, which was a gush enhancer-based autoencoder model. The vanishing gradient problems were solved by employing multiple blocks of ResNet-101 layers, and the vessel improvement filter has been integrated to improve the dehazing performance. The efficiency of the developed approach has been revealed through the simulation outcomes in terms of various measures.

Recent advances in real-time traffic sign detection and modern design techniques: The recent works have highly concentrated on implementing real-time traffic sign identification techniques. These techniques attain a trade-off between computational efficiency and detection accuracy for autonomous driving applications. The lightweight object identification models, such as YOLOv8 (Zhang H. et al., 2025; Wang et al., 2026), have been developed for minimizing the computational burden without reducing the detection performance. At the same time, the attention-based models, such as heterogeneous attention (Deng et al., 2025) and sign-aware attention (Asamoah et al., 2025), have showed high improvements in capturing the discriminative traffic sign patterns by eliminating the redundant data. In addition, the enhanced multi-scale fusion methods, such as multi-scale attention (Chen et al., 2026) and coordinate-attention fusion (Farhani et al., 2026), have highly enhanced the identification of tiny and partially occluded traffic signs. These recent improvements show that integrating lightweight models with attention-based feature learning gives an efficient solution for real-time smart transportation models. Inspired by these improvements, the suggested model combines image dehazing employing SPP-CAE, MAFF-FPN, and YOLOv9 with SAOO-PAS for achieving computationally efficient traffic sign identification under dynamic environmental scenarios.

Table 1, as discussed below, the conventional traffic sign identification methods have attained many enhancements in detection accuracy and feature extraction. However, the majority of the techniques either utilize computationally complex models. These models constrain the real-time deployment or show poor robustness under dynamic traffic conditions. Though the lightweight detection techniques enhance the inference speed, these models mostly face performance degradation when identifying small or partially occluded traffic signs. Likewise, the attention-based techniques improve the feature representation. However, these models have not resolved the image quality degradation issue created by the low-visibility conditions. These problems inspire the suggested MAFF-FPN-AYv9 model. The designed model integrates image enhancement, attention-based multi-scale feature fusion, and parameter optimization for enhancing both computational efficiency and detection accuracy for autonomous driving applications.

**Table 1:** Examines the features and difficulties of existing traffic sign detection methods.

References	Methodology	Features	Challenges
Liu et al. (2023)	UCN-YOLOv5	It achieves a higher accuracy rate, which helps in easy detection.	Due to a lower convergence rate and epoch counts, the performance is degraded.
Wang T. et al. (2024)	MMW-YOLOv5	The speed of the convergence is greater.	Its computational cost is very high due to its complicated network structure.
Wang et al. (2024b)	Vehicle-Mounted Adaptive Traffic Sign Detector	It improves detection accuracy for small objects without additional parameters.	Its memory requirements are high, which leads to a slowdown in the detection process.
Rani et al. (2024)	DLHR-TSDR	It is mainly used to remove the haze and provide a higher accuracy rate.	It has limitations in the various traffic signs in different regions.
Zhang L. et al. (2025)	TSD-DETR	It effectively achieves the discriminative features to elevate the system performance.	It causes computational complexity as the model is handling a large set of data volumes.
Kuppusamy et al. (2023)	YOLOv7, (CBAM)	It is used to improve traffic management and improve safety metrics.	The time required for detection is higher.
Zhao et al. (2024)	TSD-YOLO	It achieves high and better performances in various environments.	It leads to potential risks with high computational efficiency.
Wang et al. (2024a)	Lightweight vehicle-mounted multi-scale TSD	It is used to improve feature extraction and enhance the retention capabilities of data.	It has a limited coverage area and requires maintenance on complex hardware platforms.
Juneja et al. (2023b)	CNN with inverted wavelet	The visibility restoration performance of this model is higher than that of the existing dehazing techniques.	The generalization performance of the model needs to be improved.
Juneja et al. (2023a)	Autoencoder-Resnet-101	It effectively solves the overfitting issues. It also solves vanishing gradient issues.	The dehazing performance of the model is poor in videos with dense haze.

### 2.1 Significance of traffic sign detection and associated problems

Traffic signs give important details to drivers about the state of the roads, speed limits, and turn instructions. Autonomous cars depend on their capacity to recognize traffic signs to make decisions. This allows them to change quickly, stop at junctions, and yield when necessary. This guarantees safer mobility for drivers and pedestrians and lowers errors by humans, which have been a major contributor to traffic accidents. Effective detection systems are very important in urban environments, where traffic patterns are complex and diverse. To ensure that all cars on the road may safely coexist, they are also crucial in the integration of autonomous and human-driven vehicles. Reliable traffic sign detection will continue to be a crucial part of safe and effective driving as the need for completely automated transportation systems grows.

The major challenges related to the existing works are given as follows.

- Standard data sources are required to fetch the input data. But in earlier times, the image quality was affected due to the limited datasets. This may mislead the detection process and degrade the performance.
- The traditional approaches are unable to forecast the precise and quick detection of different traffic signs since it does not alter or extract the target object’s attributes. To address the aforementioned problems, it is necessary to employ a deep advanced feature extraction module.
- To get high multi-scale target detection accuracy, the traffic sign provides multi-scale features, which lead to better performance. Without adapting these features, it is very difficult to handle the efficiency and accuracy of the targeted object.
- Since the data falls under traffic congestion, road accidents, and unfavorable weather, an effective model is required. Thus, to enhance the image quality and the accuracy of detection, the image hazing method is recommended.

### 2.2 Proposed method

The developed method utilizes image dehazing and deep learning techniques for detecting traffic signs from real-time camera transmissions. The developed model addresses various environmental challenges such as occlusion, varying lighting conditions, and sign diversity, which ensures its robustness in real-world driving scenarios.

*Identifying the Pre-processing Integration of Image Dehazing Techniques*: The existing models may have suffered from low visibility, which has a high impact on image quality. Thus, the SPP-CAE-based image dehazing system is used in this work, which can enhance the visibility without affecting the crucial details present in the given images. It can also perform even with complex conditions by applying the features of the SPP-CAE, which increases the detection accuracy.

*Multi-Scale Feature Extraction with Attention Mechanisms*: The developed model used MAFF-FPN, which can extract features by using diverse scales; however, conventional systems are based only on the fixed-scale feature extraction ability. It can also handle the challenging backdrops by leveraging the power of the attention mechanism, thus allowing the model to focus on the essential features.

*Enhanced Object Detection with Adaptive YOLOv9*: The adaptive YOLOv9 uses the power of the existing YOLOv9 with optimized parameters for the optimal solution, resulting in better and enhanced ability to capture the distinctive traffic sign features. By using the adaptive modification, YOLOv9 outperformed the existing detection models.

*Robustness across Diverse Datasets:* This work used two significant databases, and they are BDD100K and TT100K, to evaluate the effectiveness of the model. Using these datasets proved the model’s good performance in terms of durability and enhanced generalization.

*Practical Relevance for Autonomous Systems*: The suggested approach is used to handle practical problems in the ITS sector. By leveraging the image enhancement system, multi-scale components, and optimization, the developed model proved its real-time applications in autonomous cars.

## 3 Overview of the developed traffic sign detection model with associated existing problems and image collection details

### 3.1 Overview of the developed model

The developed model used multi-scale feature extraction ability with pre-processing to improve the accuracy and robustness when detecting the traffic signs for the safety of the drivers. The developed model can solve the problems that are encountered in the conventional systems, including poor image quality, fog, haze, etc. By using the huge dataset, the developed model provides an enhanced ability to detect the actual traffic signs even with complicated traffic patterns. The required images are collected from the datasets and given to the image dehazing phase. This step is crucial in the developed model to improve the image quality, where the SPP-CAE is used for this process. This also helps in preventing the images from adverse and extremely bad weather conditions, while improving the image quality and detection accuracy. Once the images are dehazed using the SPP-CAE, they are given to the MAFF-FPN-AYv9 for detecting the traffic signs. Here, the MAFF-FPN helps to detect the object at varying scales, thus improving the ability of the model to detect the traffic signs with enhanced image resolution.

The AYv9 is employed to ensure that the model can detect the roadway marking more accurately under dynamic circumstances. Moreover, by leveraging the AYv9, the developed model can adapt to different roadway patterns and identify the traffic signs more precisely, thus improving the effectiveness while reducing the computational cost. To further enhance the performance, the SAOO-PAS is applied, which helps in optimizing the significant parameters, including hidden neurons, learning rate, and activation function. Finally, the developed model provided the detected outcome by leveraging the power of enhanced optimization, multi-scale feature extraction, and image dehazing, resulting in a robust and optimal model, which makes it appropriate for real-time use in autonomous driving and ITS applications.

### 3.2 Performance of proposed model with the existing hybrid methods

The developed model outperforms the existing hybrid systems when it comes to feature learning ability. The existing hybridized systems have the complexity in handling the scale fluctuations, retaining the enhanced robustness and the recognition of tiny and occluded indicators. However, the developed model can solve all these problems by leveraging the enhanced optimization algorithm with a deep learning model. The following is a full explanation of how our technology can produce more satisfactory traffic sign detection results. The network can handle images with different levels of detail more effectively by using the SPP structure to extract features from the image at numerous scales. This method successfully eliminates the image’s haze when used in conjunction with the CAE, improving the crucial elements that the detection model must concentrate on and restoring visibility. Due to environmental changes like fog, rain, or other atmospheric disturbances, which can obscure or distort signals, this dehazing technique is very important for traffic sign identification. The multi-scale extraction of features is essential for differentiating both large and small signs for traffic, whether on wide roads or in urban environments with numerous backdrop objects.

**Figure 1:**
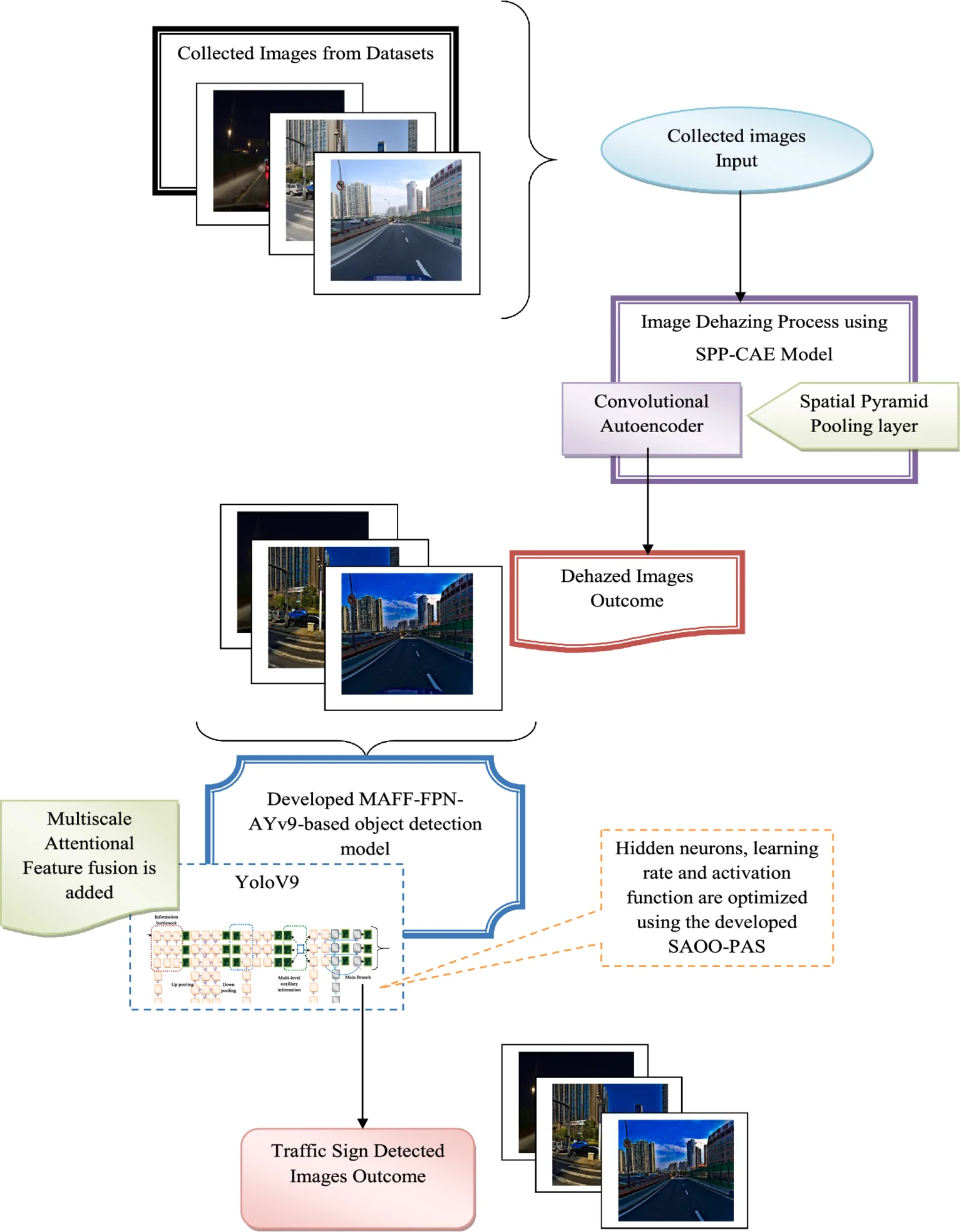
Structural depiction of the developed traffic sign detection approach (images reproduced with permission from the BDD100K dataset, https://www.kaggle.com/datasets/awsaf49/bdd100k-dataset, licensed under CC0, and the TT100K dataset, https://www.kaggle.com/datasets/braunge/tt100k, licensed under CC-BY 4.0).

The MAFF-FPN allows the network to focus on the most relevant data for sign identification by combining features from multiple layers into a single accountability. To ensure that the design can effectively recognize roadside markings in a variety of situations, the AYv9 object recognition technology is also employed. The well-suited and successful YOLO model for instantaneous object detection has improved speed and accuracy in version 9. By including population modification updates into the optimization process, the SAOO-PAS technique ensures that the framework is not only developed for overall efficiency but also prepared to adapt to specific traffic sign-detecting scenarios that can arise in real-world settings. This method leads to more precise detection capabilities, especially in complex road situations or challenging environmental conditions. Figure 1 shows the structural representation of the designed traffic sign detection model.

Key observations and insights of the proposed work:

*Standard image collection for effective validation:* The significant images are collected from the standard data sources. It helps to assess the suggested model’s generalization capacity.

*Image dehazing improves the detection process:* The images utilizing SPP-CAE minimize the weather-based and haze distortions. The clear images result in highly accurate feature extraction, most importantly for the distance or tiny traffic signs.

*MAFF-FPN improves the feature representation:* The designed MAFF-FPN captures the relevant features at distinct scales, thus improving the identification of traffic signs in distinct sizes. The attention methods allow the technique to concentrate on the related regions while eliminating the unwanted background noise and enhancing the model’s performance.

*Optimized YOLOv9 improves the performance rate:* By employing the SAOO-PAS, the YOLOv9 parameters are optimized. It guarantees optimal identification thresholds and better traffic sign localization. The designed SAOO-PAS results in better improvements over the existing performance of the YOLOv9 model.

*Integration of promising models improves the accuracy rates:* The integration of image dehazing, MAFF-FPN, and optimized YOLOv9 provides higher accuracy than employing any individual module alone. This unified framework is very efficient under complex environmental conditions.

### 3.3 Implemented SAOO-PAS

To optimize the AYv9 method’s parameters and guarantee efficient and precise object recognition, the SAOO-PAS implementation is essential. An improved variant of the AOOA, SAOO-PAS, is modelled, which leverages the honey bees’ natural foraging habits. By itself, AOOA simulates the competitive and cooperative interactions that occur within a bee colony, where members exchange data and collaborate to discover the best solutions. The queen is essential to strike a balance between exploitation and exploration in traditional AOOA (Al-Sharqi et al., 2024). In this case, the population is determined by the apiary using Equation 1.


CR=S+0.1×f+0.9×y (1)

The population of the apiary is represented by the term CR in Equation 1, the best solution selected as the queen is *S,* the maximum worker age is *f,* and the total number of workers is *y*. However, it only has one queen, or about 10% (0.1) of the drones, and 90% (0.9) of the workers are static, which may result in less-than-ideal performance. The algorithm may prematurely converge on less-than-optimal solutions if it is static and does not sufficiently explore the solution space.

In this context, *W_r_F* stands for best fitness and *C_r_F* for current fitness. In AOOA, Equation 1 is updated and expressed in Equation 2 to resolve the problems and improve convergence and parameter optimization. A more balanced and efficient optimization process is performed by SAOO-PAS’s capacity to modify 0.1 of drones and 0.9 of workers. SAOO-PAS is utilized in the AYv9 model to optimize important hyperparameters that affect the model’s effectiveness.


$$\mathrm{CR}=S+\frac{\mathrm{CrF}}{{\left(\mathrm{WrF}\right)}^{2}}\times f+(1-\frac{\mathrm{CrF}}{{\left(\mathrm{WrF}\right)}^{2}})\times y$$ (2)

**Figure 2:**
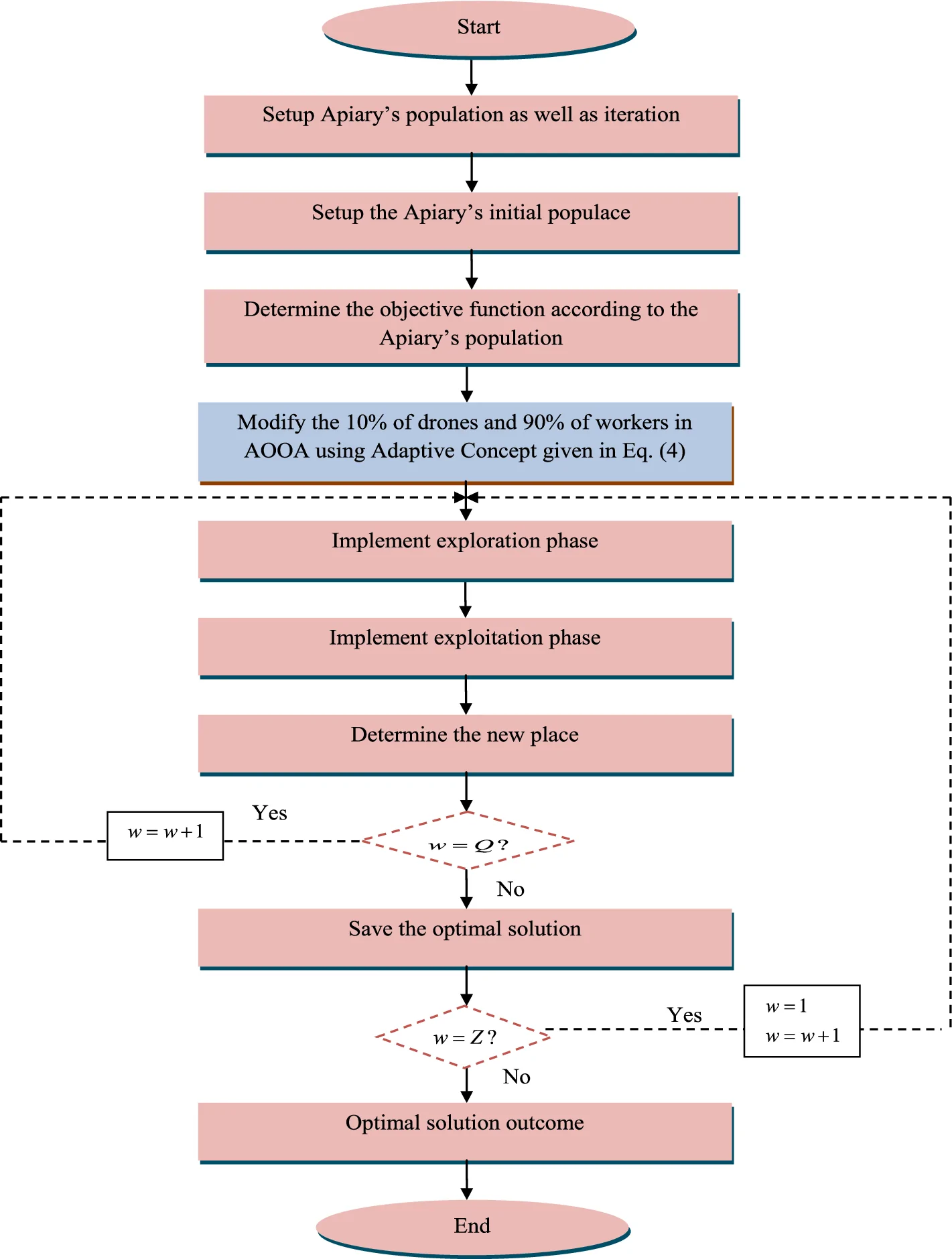
Flowchart of the proposed SAOO-PAS.

Here, the term *C_r_F* is the current fitness, and *W_r_F* is the best fitness. To solve the issues and improve convergence and parameter optimization, Equation 1 is replaced by Equation 2 in AOOA. The ability of the developed SAOO-PAS to modify the 0.1 of drones and 0.9 of workers, which may result in balanced and optimal optimization results. Moreover, fine-tuning the parameters of AYv9 using the SAOO-PAS also has a greater impact on enhanced model efficiency. Then, the proposed SAOO-PAS also reduced the false negatives and positives by using the enhanced and updated adaptive concept given in Equation 2, which plays a vital role in safety-sensitive applications. It also improves the effectiveness of the optimization process by speeding up the convergence ability to further strengthen the robustness through encouraging and selecting only the optimal set of parameters. Finally, the optimization parameters using the SAOO-PAS allow the developed model to perform well in real-time applications. Thus, this paves the way to support the design and development of robust autonomous vehicle technologies by ensuring that the system can handle the complicated dynamic settings, along with the given fluctuation present in the operational situations. Figure 2 shows the flowchart for the suggested SAOO-PAS.

## 4 Image pre-processing for dehazing using spatial pyramid pooling-based model with developed population amendment strategy

### 4.1 Description of CAE model

The main benefit of employing CAE for removing hazing is its capacity to detect and eliminate undesired distortions, such as blur, fog, or haze, while maintaining critical image features that are essential for the recognition of objects. A convolutional layer, encoder, and decoder are some of the components that make up the CAE (Yagis et al., 2021). The CAE’s encoding learns its shape from the input data. The decoder components use the latent representation to recreate the input, making it appear to be the original data. While determining the function, the CAE reduces the restoration uncertainty between the input and output. Equation 3 (Yagis et al., 2021), which is explained by the decoding component of the organization, reconstructs the data that was provided after it has been sent to the encoding module related to the CAE.


$${\bar{b}}_{m}=k(j(b_{m}))$$ (3)

The term $b_{m}$ is the input image, ${\bar{b}}_{m}$ is the reconstructed image, $j(b_{m})$ is the function of the encoder, and *k* is the kernel size. Eq. 4 expresses the CAE’s loss coefficient.


$$P=\frac{1}{R}=\sum_{M}{\left(b_{m}-k(j(b_{m}))\right)}^{2}$$ (4)

The term *P* is the loss function, and *R* is the regularization factor. The stage of convolution is followed by the sequential data normalization. The downsampling process is performed by the max-pooling function, which is situated between the two convolutional modules. The convolutional layer, normalization, and Rectified Linear Unit (ReLU) functions make up the decoder components.

Training details of CAE: The CAE technique is trained before detection by employing paired hazy and clean images. The technique is optimized by employing the Adam optimizer with a fixed learning rate of 0.001 for 50 epochs. Mean Squared Error (MSE) loss with L2 regularization is utilized. Early stopping is employed when the validation loss does not improve for 10 epochs. Table 2 shows the training hyperparameters of CAE.

**Table 2:** Training hyperparameters of CAE.

Parameter	Value
Epochs	50
Batch size	16
Optimizer	Adam
Learning rate	0.001
λ (Regularization)	0.0001
Loss	MSE + L2
Input size	256 × 256

### 4.2 Image dehazing using SPP-CAE

The method of image dehazing is essential for improving the quality of images affected by unfavorable surroundings, which often mask significant imagery. Image dehazing is used to improve the quality and contrast of the images to easily recognize the traffic sign language and colors. This has become more important since autonomous vehicles are mostly based on high-quality images, which aid in making adjustments in real-time road safety applications. Image dehazing further improves the image contrast between the traffic signs and the background, paving the way to provide accurate detection even in complex traffic situations. Thus, this work developed SPP-CAE for the image dehazing process to achieve better detection results.

Reasons for selecting SPP-CAE: The SPP-CAE model is selected in this work for performing the image dehazing process. This model is selected after considering its computational efficiency and restoration efficiency for the traffic sign identification process. The existing image dehazing models (Lyu et al., 2026; Jin et al., 2026) are based on handcrafted image enhancement methods. These models mostly struggle to secure the fine structural information of tiny traffic signs. Also, normally, these models show poor robustness under varying weather conditions. Though recent deep learning-aided dehazing models (Huang et al., 2025; Liang et al., 2026) attain high restoration quality, most of the methods utilize complex models with high memory and computational demands. These features make the existing models less suitable for real-time smart transportation devices. On the other hand, the suggested SPP-CAE integrates the CAE’s representation capacity with SPP’s multi-scale contextual learning. This integration allows efficient haze removal while securing significant traffic sign patterns. Hence, the designed SPP-CAE offers a better balance among computational complexity and image restoration quality. Hence, it enhances the performance of the subsequent traffic sign identification process.

**Figure 3:**
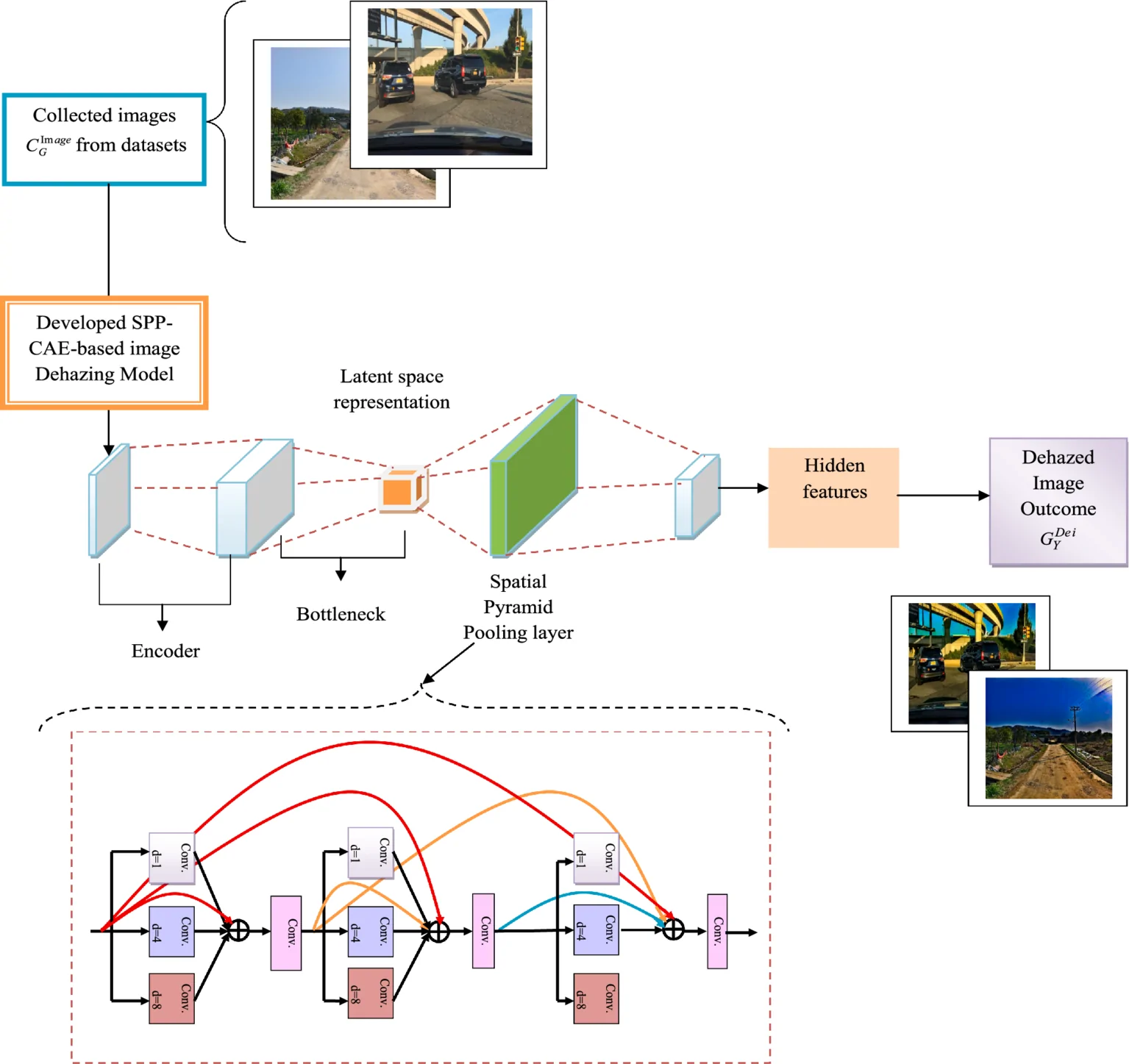
Structural illustration of the developed SPP-CAE-based image dehazing model (images reproduced with permission from the BDD100K dataset, https://www.kaggle.com/datasets/awsaf49/bdd100k-dataset, licensed under CC0, and the TT100K dataset, https://www.kaggle.com/datasets/braunge/tt100k, licensed under CC-BY 4.0).

Image quality restoration has emerged as a promising environmental aspect, and it is the main aim of the image dehazing system in the developed SPP-CAE model, which helps in preparing the images for further detection processes. Initially, the collected images are given to the CAE. Here, the encoder block present in it helps in compressing the images to create a low-dimensional feature, while the decoding block then reconstructs the image to its initial form. Then, the SPP is added to this to improve the spatial resolution of the images. It can also combine the features at various levels by using its multi-scale feature extraction ability when processing the images, which have different sizes and more objects. Depending on how far away the camera is from the sign and how the camera is oriented in relation to the sign, signs may look various sizes. The network can effectively pool and process features at different scales by using SPP, which enables it to better manage the different sizes and locations. The combination of SPP and CAE improves the overall ability of the system to deal with complex, distorted images by capturing both local and global features at different scales. Moreover, CAE’s dehazing ability ensures that significant details such as text, colors, and shapes on traffic signs are preserved and enhanced, allowing the detection model to identify them with higher precision. Figure 3 shows the structural illustration of the developed SPP-CAE-based image dehazing model.

Architecture details of SPP-CAE: The designed SPP-CAE includes a three-layer convolutional encoder, followed by a Spatial Pyramid Pooling (SPP) and a symmetric decoder. The encoder extracts the multi-scale haze-based features, whereas the SPP captures the contextual data at different spatial resolutions to improve the dehazing performance. Table 3 gives the architecture details of the designed SPP-CAE.

**Table 3:** Architectural details of the designed SPP-CAE.

Layer	Type	Filters	Kernel	Stride	Pooling
Encoder-1	Conv + ReLU	64	3 × 3	1	MaxPool 2 × 2
Encoder-2	Conv + ReLU	128	3 × 3	1	MaxPool 2 × 2
Encoder-3	Conv + ReLU	256	3 × 3	1	MaxPool 2 × 2
SPP	Pooling	–	{1 × 1, 2 × 2, 4 × 4}	–	–
Decoder-1	Deconv + ReLU	128	3 × 3	1	UpSample
Decoder-2	Deconv + ReLU	64	3 × 3	1	UpSample
Output	Conv	3	3 × 3	1	–

Trade-offs and Limitations of SPP-CAE: Though the designed SPP-CAE gives more advantages, it gives some trade-offs. The inclusion of the dehazing process poses additional pre-processing time before object identification. It leads to a modest increase in the overall computational expenses. In addition, though the designed SPP-CAE works effectively under adverse weather conditions, the high atmospheric degradation may still minimize the image restoration quality contrasted to the huge state-of-the-art dehazing models that leverage transformer-aided techniques. However, these techniques normally demand high computational memory and resources. Hence, SPP-CAE is selected because it gives a practical balance among computational efficiency and restoration performance with the suggested traffic sign identification model.

## 5 Proposed multiscale attentional feature fusion-based network and adaptive mechanism for object detection with optimized parameters

### 5.1 MAFF-FPN

To improve the overall efficiency in tasks like object recognition, a neural network architecture known as MAFF-FPN uses the idea of the Feature Pyramid Network (FPN) for extracting features at multiple levels. However, it improves the feature fusion process by adding an attention mechanism to deliberately focus on the most relevant data of various sizes. FPN has been shown to successfully improve the abilities of deep learning systems to handle object identification tasks, predominantly for identifying objects of different sizes. It is initially suggested to solve the hierarchical feature fusion problem of CNNs. The descriptive power inherent in the feature pyramid is now increased by the usage of this to promote data adaptation and enhance feature propagation. To effectively utilize multi-scale features, FPN combines bottom-up networks with top-down networks to create simultaneous cross-scale relations.

Later, FPN is applied to the head for multi-scale feature integration. To accomplish the top-down unification of features, FPN first captures the feature maps from the CNN systems and then applies upsampling and granular map features. To satisfy the requirements of feature combination at multiple levels, it is intended to swiftly integrate patterns into various types and improve the result of feature combining via the addition of more layers. This allows the model to treat low-level spatial data alongside semantic data at the highest level equally throughout the head section.

Attention mechanism used in MAFF-FPN: The attention mechanism in the designed MAFF-FPN is implemented to improve the multi-scale feature fusion by capturing the related features and also by eliminating the unwanted background responses. In an existing FPN, the feature maps from different pyramid levels are integrated by employing simple element-wise addition. It considers all the spatial and channel regions ideally. The fusion task focuses on the partially occluded, tiny, and added to the complex backgrounds. To address this issue, the MAFF-FPN includes a hybrid attention mechanism, which includes spatial attention and channel attention at each pyramid level. The channel attention captures the relevant feature channels via max pooling and global average pooling. On the other hand, the spatial attention underscores the significant spatial areas by focusing on the relevant regions. These features from different scales are further integrated. It allows the model to improve the detection accuracy for the tiny signs.

### 5.2 YOLOv9

YOLOv9 (Rizzieri et al., 2024) is a sophisticated object detection algorithm that uses cutting-edge approaches such as programmatic gradient data and a generalized efficiency layer aggregation network.

The YOLOv9 model addresses the main problem in algorithms for deep learning, such as memory degradation throughout the network’s layers. The reversible factors and the informed bottleneck hypothesis are the two main breakthroughs of the YOLOv9 theory. It demonstrates how crucial details are lost throughout the data transportation phase at subsequent tiers. Eq. 5 provides a mathematical description for this phenomenon


$$P(D,D)\geq P(D,s_{\upsilon }(D))\geq P(D,k_{\lambda }(s_{\upsilon }(D)))$$ (5)

The variables that constitute the parameters of adjustments act *k* and *s* are denoted by the characters λ and υ . The inputs are represented as *P* and *D* are transformed data. Suboptimal convergence and ineffective updates of gradients may result from such data loss. Eq. 6 (Rizzieri et al., 2024) defines a reversible function as one that may be reversed without losing any kind of data.


$$D=u_{\lambda }(i_{\alpha }(D))$$ (6)

The inverted and reversible function’s constants are shown here as λ and α , respectively. All input data is maintained across the network’s levels thanks to this method. With its fast speed of computation and precision in detection, YOLOv9 is highly adaptable to a wide range of tasks because of its design, which allows customizable incorporation of various mathematical components.

### 5.3 Proposed MAFF-FPN-AYv9-based object detection model

The proposed MAFF-FPN-AYv9-based object detection model has made substantial progress in real-time object and traffic sign identification, particularly when considering autonomous automobiles and connected vehicles. The developed model leverages the power of some cutting-edge systems, which help in addressing the complex problems present when detecting traffic signs. The integration of the MAFF-FPN and AYv9 is a significant element used in the developed system to provide scalable, effective, and dependable results in detecting the roadway markings. To tackle the existing issues in handling images, even in the presence of occluded conditions and low-contrast images, has become the main objective of the developed MAFF-FPN-AYv9-based traffic sign recognition model. The existing models suffer from providing optimal results due to the variation in size, occlusion, and obscuration of the images. The developed model can solve these problems by incorporating MAFF-FPN and AYv9 with the enhanced optimization algorithm. By using these components, the proposed model improves detection performance even in complex situations. In the developed MAFF-FPN-AYv9-based traffic sign recognition model, MAFF-FPN has become one of the advanced components. The dehazed images are initially given to this element. The FPN component can create the feature pyramids by gathering the features at various scales, thus making it a versatile option for detecting objects. It also allows the proposed model to deeply analyze the images from fine-grained to coarse features by capturing the smaller items.

However, the FPN has a complexity in detecting the objects more accurately, since the images have different sizes and positions. Mainly, it is challenging to detect when the traffic signs are located farther away or when they are occluded by other objects. Thus, the attention feature element is added as another innovative element into the proposed system. By using the power of the attention mechanism, the MAFF-FPN aids in improving the standard FPN, as it allows the model to focus only on more essential regions, which is required for optimal detection. The proposed system can allocate more weights to the relevant features by leveraging the attention system, while neglecting the irrelevant features from the background of the objects. Moreover, the model’s accuracy can also be improved by using this attention system, as it can differentiate the traffic signs from other objects more effectively. Finally, by analyzing the object at different scales, it improves the capacity of the model to capture both the huge and tiny details, even the presence of occluded objects.

**Figure 4:**
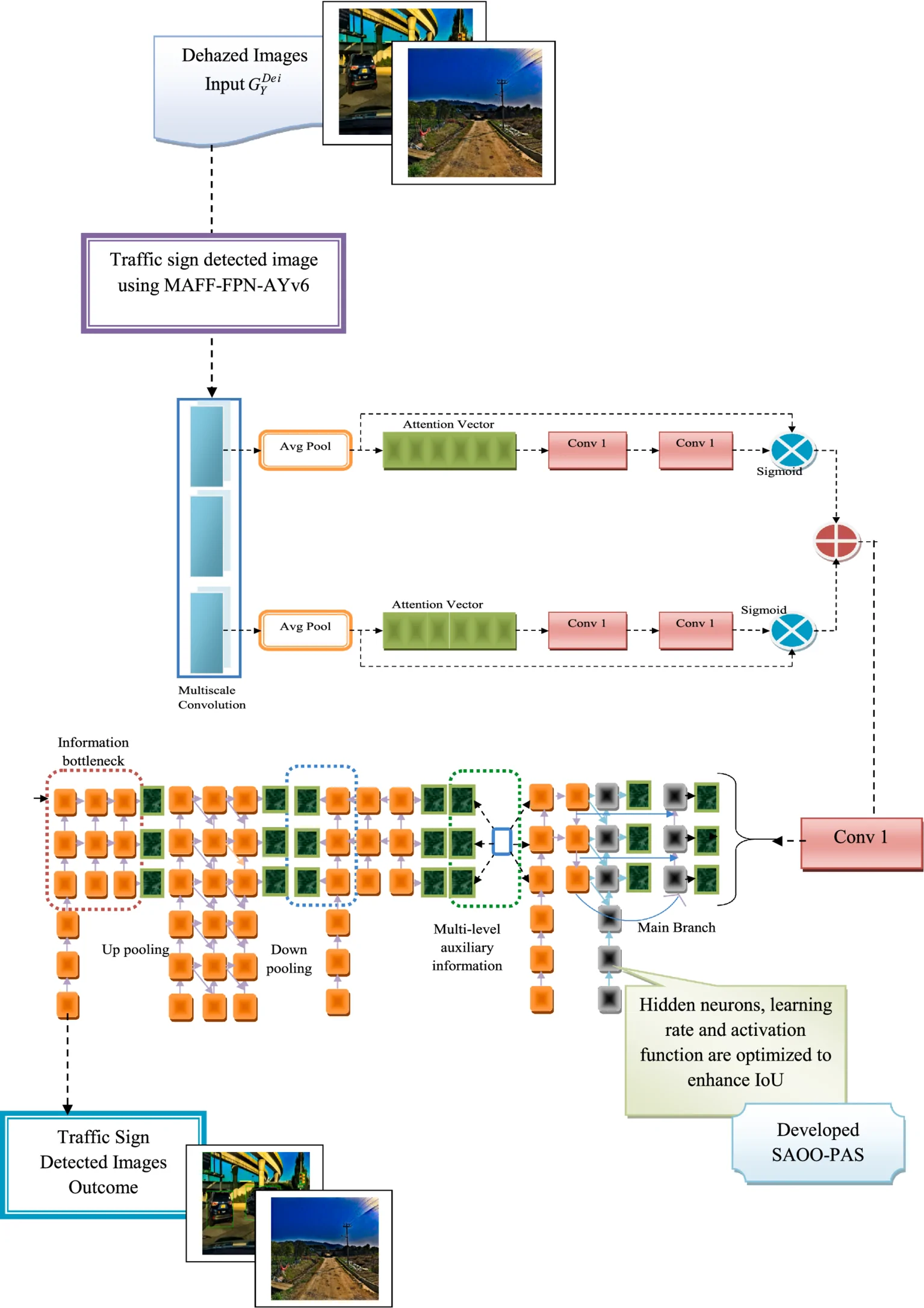
Diagrammatic depiction of the proposed MAFF-FPN-AYv9-based traffic sign detection model (images reproduced with permission from the BDD100K dataset, https://www.kaggle.com/datasets/awsaf49/bdd100k-dataset, licensed under CC0, and the TT100K dataset, https://www.kaggle.com/datasets/braunge/tt100k, licensed under CC-BY 4.0).

Once the images are processed by using the MAFF-FPN, they are given to the AYv9 for the final detection process. YOLOv9 is an effective system that is famous for tasks like object detection. By sectioning the images into a grid, the YOLOv9 initially detects the boundaries, along with the classes of each grid. The performance of this YOLOv9 is improved by the adaptive parameters, which are optimized by using the SAOO-PAS. It allows the proposed model to adapt to the dynamic environmental conditions by optimizing the parameter set. The YOLOv9 hyperparameters, including activation function, hidden neurons, and learning rate, are tuned by the proposed SAOO-PAS, which improves the Intersection over Union (IoU) and makes the model more flexible. The diagrammatic depiction of the proposed MAFF-FPN-AYv9-based traffic sign detection model is given in Figure 4.

*Objective Function*: Equation 7 provides the objective function of the designed MAFF-FPN-AYv9-based object detection model. The YOLOv9 technique may face overfitting problems during the training phase, which may degrade the model’s performance. To improve YOLOv9’s detection performance, the hyperparameters of the YOLOv9 technique are optimally selected with the help of the SAOO-PAS. The SAOO-PAS algorithm is effective in providing optimal solutions, even under complex scenarios, like parameter optimization in deep models. By optimally selecting the hyperparameters of the YOLOv9 technique, the designed SAOO-PAS maximizes the IoU rate. Therefore, Equation 7 serves as an effective objective function for guiding the model towards achieving high-quality object detection results in challenging real-world conditions.


$$\text{OFun}=\underset{\left\{A_{S}^{\text{Yolo}V_{9}},H_{G}^{\text{Yolo}V_{9}},L_{Q}^{\text{Yolo}V_{9}}\right\}}{\text{argmin}}(\frac{1}{\mathrm{IoU}+\in })$$ (7)

Here, the terms $A_{S}^{\text{Yolo}V_{9}}$ , $H_{G}^{\text{Yolo}V_{9}}$ and $L_{Q}^{\text{Yolo}V_{9}}$ are the activation function, hidden neurons, and learning rate, and they are selected in the range of [1,5], [5, 255], and [0.01, 0.99]. The expression for IoU is given in Equation 8. Table 4 displays the optimized YOLOv9 model’s hyperparameter details. The term ∈ specifies a small positive constant. Here $\in =10^{-6}$ . When the IoU becomes zero, numerical instability occurs. To eliminate this, the positive constant ∈ is included with the objective function. With this formulation, the cost function remains stable across the IoU ranges.

**Table 4:** Optimized hyperparameter details of YOLOv9.

Optimized hyperparameters of YOLOv9			
Hyperparameter	Symbol	Search range	Reason for optimization
Activation function	$A_{S}^{\text{Yolo}V_{9}}$	{ReLU, Leaky ReLU, SiLU, Mish, ELU} (1–5)	Controls non-linearity and feature discrimination
Number of hidden neurons	$H_{G}^{\text{Yolo}V_{9}}$	[5, 255]	Affects model capacity and feature representation
Learning rate	$L_{Q}^{\text{Yolo}V_{9}}$	[0.01, 0.99]	Strongly impacts convergence speed and stability

IoU: An object-detection system’s accuracy is determined using the IoU metric.


$$\mathrm{IoU}=\frac{|C\cap D|}{C\cup D}$$ (8)

Here, the terms C∩D and C∪D are the area of intersection and union between the predicted and ground truth bounding boxes, and *C* and *D* represent the predicted area (mask) and ground truth area (mask).

Justification of objective function formulation: In the suggested traffic sign identification approach, the main performance measure for localizing the bounding box is the IoU. Since IoU estimates the overlap between the ground truth and predicted bounding boxes, the high IoU values indicate better identification performance. Hence, the IoU is a maximization measure. Nevertheless, the designed SAOO-PAS algorithm works under a minimization-aided optimization model. To guarantee the compatibility between the optimization and the detection, the objective function is derived as a minimization of the inverse of IoU. By converting the IoU maximization into the minimization of its inverse, the optimization problem becomes more consistent with the designed SAOO-PAS mechanism.

Incorporation of inverse-IoU objective into the SAOO-PAS optimization process: During each SAOO-PAS algorithm’s iteration, the following operations are performed. Initially, a candidate solution’s population is produced. Each member specifies hyperparameters, such as hidden neuron counts, activation function, and learning rate. On the training set, the YOLOv9 technique is trained for each member. Further, the detection operation is conducted during the validation phase. For each detected object, the IoU is estimated, and then the average IoU across all validation samples is employed to estimate the inverse IoU cost of the candidate. The designed SAOO-PAS further employs stochastic search exploration and exploitation methods to iteratively reduce this cost across the iterations and population. By reducing the inverse IoU cost, the SAOO-PAS efficiently maximizes the detection IoU. It also assists the hyperparameter search towards configurations that provide high identification functionality.

IoU computation during optimization process: The IoU is estimated during the SAOO-PAS optimization operation by employing the bounding box overlap formulation. Here, the intersection region between the specific ground truth and forecasted bounding box annotations is partitioned by their union area. The IoU is not estimated on a training set or a held-out test during optimization.

The training test is highly employed for the model weight learning under the hyperparameter configuration. The test set is utilized for final performance reporting to guarantee unbiased validation.

Optimization scope of SAOO-PAS: In the suggested work, the designed SAOO-PAS is employed as an offline parameter tuning module before final model training. The SAOO-PAS is not added within a gradient-aided backpropagation task, and in real-time, it does not upgrade the network weights. Instead, the designed SAOO-PAS works externally to the learning strategy to estimate the optimal hyperparameter configuration prior to the last training stage starting. The overall training process includes four phases. Initially, the dataset is partitioned into test, validation, and training subsets. Secondly, the designed SAOO-PAS performs offline hyperparameter optimization. Here, the member configurations are produced, and the technique is trained for a specific number of epochs utilizing the training set. Further, the validation of IoU is estimated and employed as the fitness factor to estimate each candidate. The designed SAOO-PAS selects the optimal hyperparameters through iterative population updates. Thirdly, the YOLOv9 technique is trained by employing the optimally selected hyperparameters. Lastly, the trained technique is validated. The designed SAOO-PAS tunes only the network hyperparameters, not the architectural structure. Most importantly, the implemented SAOO-PAS optimizes the YOLOv9 model parameters, such as learning rate, activation function, and the number of hidden neurons. Thus, the designed SAOO-PAS improves the detection performance through optimal parameter selection.

Computational overhead during SAOO-PAS-based parameter optimization: The designed SAOO-PAS algorithm is utilized only during the offline hyperparameter optimization phase. Also, it does not involve real-time inference. Hence, its computational overhead affects only the training time, not the runtime identification speed. Table 5 gives the configuration, training time, and inference latency analysis of the designed SAOO-PAS. From Table 5, though the SAOO-PAS increases the overall training time, the overhead is offline and one-time. After the optimal hyperparameters are selected, the designed SAOO-PAS is eliminated from the framework, and the AYv9 technique works as a standard YOLOv9 with no specific variation in the inference latency. Hence, the suggested approach maintains real-time feasibility while achieving improved detection performance.

**Table 5:** Configuration, training time, and inference latency analysis.

Configuration of SAOO-PAS	
Parameter	Value
Population size	10
Number of decision variables	3
Maximum iterations	50
Total fitness evaluations	500
Hardware	NVIDIA GeForce RTX 4050 GPU, Intel i5-13420H

Training time analysis	
Training configuration	Average training time
Standard YOLOv9 (no optimization)	~3.8 h
YOLOv9 + SAOO-PAS (tuning phase)	~5.1 h
Relative overhead	≈ 34% increase

Inference latency analysis	
Model	Inference Time (sec/image)
YOLOv9	~0.26
SAOO-PAS–MAFF-FPN–AYv9	~0.28

## 6 Results and discussion

### 6.1 Simulation setup

In this study, optimization was performed using a population-based method with the SAOO-PAS in Python, where the number of populations of the SAOO-PAS was set to 10, the chromosome length of the SAOO-PAS was 3, and the total iteration of the SAOO-PAS was set to 50. The developed model used the processor named Intel(R) Core (TM) i5-13420H CPU @ 2.10GHz,16.0 GB RAM with a 64-bit operating system, x64-based processor, and it has the Windows 11 Pro Edition and Python 3.12 Interpreter. Finally, the CPU was an Intel Core i5-13420H @ 2.10 GHz, and an NVIDIA GeForce RTX 4050 GPU.

To assess the effectiveness of this optimization, comparisons were made with several state-of-the-art optimization algorithms, including the Pine Cone Optimization Algorithm (PCOA) (Valikhan Anaraki and Farzin, 2024), Red-Tailed Hawk Algorithm (RTH) (Ferahtia et al., 2023), Lotus Effect Optimization Algorithm (LEA) (Dalirinia et al., 2024), and Apiary Organizational-Based Optimization Algorithm (AOOA). The performance of the suggested model was further compared across multiple object detection models, such as YOLOv3 (Xiong et al., 2023), MMW-YOLOv5, YOLOv7, and YOLOv9 (Kang et al., 2024). The effectiveness of each model was assessed according to its resilience in various situations, processing rate, and precise detection.

### 6.2 Dataset description

Dataset-1 (BDD100K Dataset): The images are taken from the link “https://www.kaggle.com/datasets/awsaf49/bdd100k-dataset”. A representative subset including 2,763 images with 10 traffic sign classes is employed in this work. This dataset was gathered from real-driving environments and includes various traffic scenarios captured under daytime and nighttime settings, rain, fog, cloudy weather, dynamic illumination, motion blur, shadows, and partial occlusions. These various environmental scenarios make the data source applicable for estimating the efficiency of the traffic sign identification models under real-time autonomous driving scenarios. The raw data images are in the dimension of 512 × 512 × 3_,_ and the images still maintain the same dimension 512 × 512 × 3 after applying the dehazing process. Similarly, after the object detection step, the images remain at 512 × 512 × 3, ensuring consistency in the feature dimensions throughout the process.

Dataset-2 (TT100K Dataset): The images are taken using the link “https://www.kaggle.com/datasets/braunge/tt100k”. A subset including 3,069 images specifying 49 traffic sign types is employed. This data source includes traffic scenes gathered from original urban roads with specific changes in partial occlusions, background complexity, object scale, viewing angles, traffic density, and illumination. Though the data source mainly concentrates on the diversity of traffic signs rather than adverse weather, it still gives specific changes in real-world driving conditions. Like Dataset 1, the raw data images are initially sized at 512 × 512 × 3_._ After the dehazing step, the dimensions of the images are unchanged, remaining at 512 × 512 × 3. The detected images, post-object detection, also retain the same dimensions 512 × 512 × 3.

The selected data sources include a huge range of real-world driving scenarios, such as changes in weather conditions, traffic density, camera viewpoints, illumination, and object scale. It allows the detailed validation of the recommended model. Especially, the inclusion of low-light, foggy, rainy, and blurred scenes in the BDD100K dataset gives a specific benchmark for validating the efficiency of the designed SPP-CAE dehazing strategy under degraded visibility scenarios. These dynamic scenarios resemble the real-time autonomous driving scenarios where accurate traffic sign identification is important.

### 6.3 Dataset subset justification and analysis

Though the full BDD100K data source is huge, training on the overall dataset is computationally complex because of the high training time and high-resolution inputs. Hence, a representative subset is selected in this work. To guarantee that this reduction does not present sampling bias or impact the generalization of an approach, additional experiments are conducted by employing increasing dataset sizes with detailed statistical validation.

*Effect of dataset size on performance:* To validate the impact of the dataset size, the validations are performed utilizing 2.7, 5, 10, and 20% of the BDD100K data source. Each analysis is repeated five times to form stochastic changes during training. The results in Table 6 elucidate that increasing the dataset size results in marginal enhancements in performance (<0.6%). In addition, the overlapping confidence intervals suggest that the improvements are not substantial.

**Table 6:** Effect of dataset size on detection performance.

Dataset %	Accuracy Mean (%)	Std Dev (σ)	95% Confidence Interval
2.7%	96.12	0.42	±0.37
5%	96.31	0.39	±0.34
10%	96.48	0.36	±0.31
20%	96.63	0.33	±0.29

**Figure 5:**
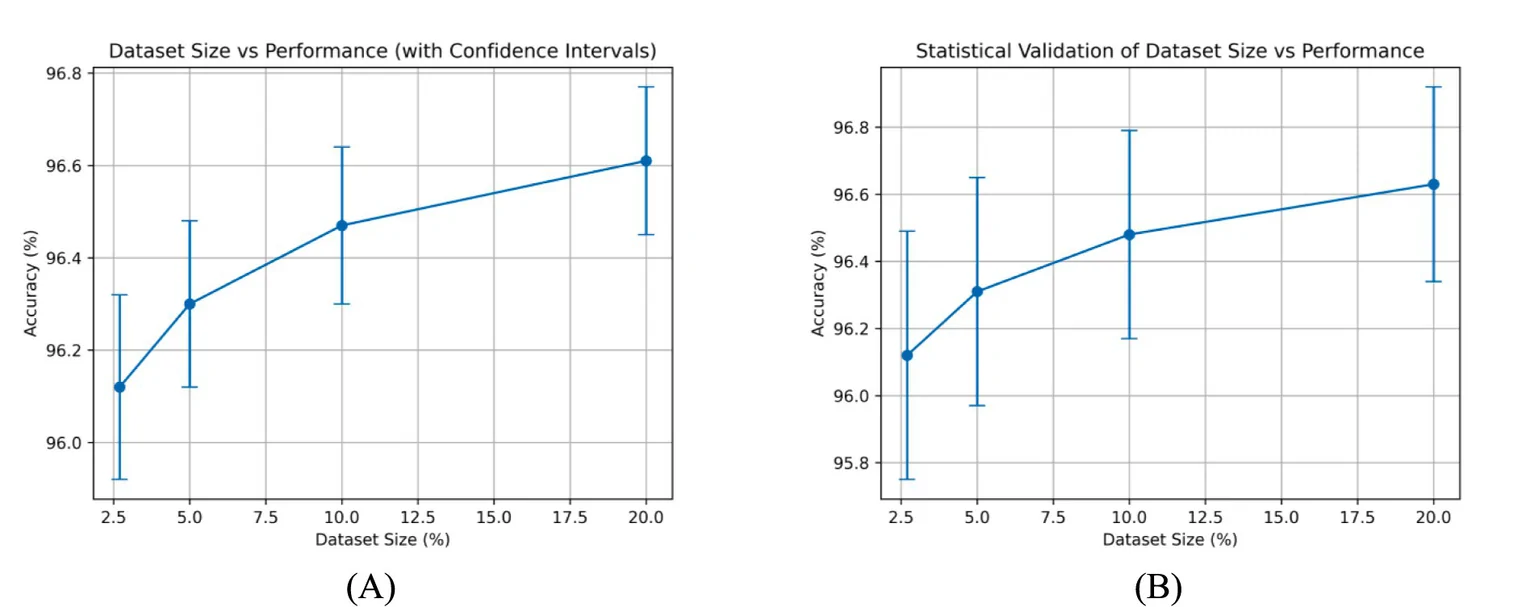
Effect of dataset size on detection accuracy with **(A)** 95% confidence intervals and **(B)** statistical validation of dataset size.

*Effect of dataset size on detection accuracy with 95% confidence intervals and statistical validation:* Figure 5 displays the effect of dataset size on detection accuracy with 95% confidence intervals and statistical validation of dataset size. As shown in Figure 5, the detection accuracy gradually improves as the size of the dataset grows from 2.7 to 20%. Nevertheless, the enhancement is very small, with accuracy increasing from 96.12 to 96.63%. Also, the results show that the 95% confidence intervals for distinct data source sizes overlap. It represents that the performance changes are not substantial. Also, a paired t-test is performed between the 2.7 and 20% datasets. The obtained *p*-value (0.18 > 0.05) indicates that the performance enhancement is not statistically significant. It recommends that increasing the size of the dataset does not result in a gain in accuracy.

**Figure 6:**
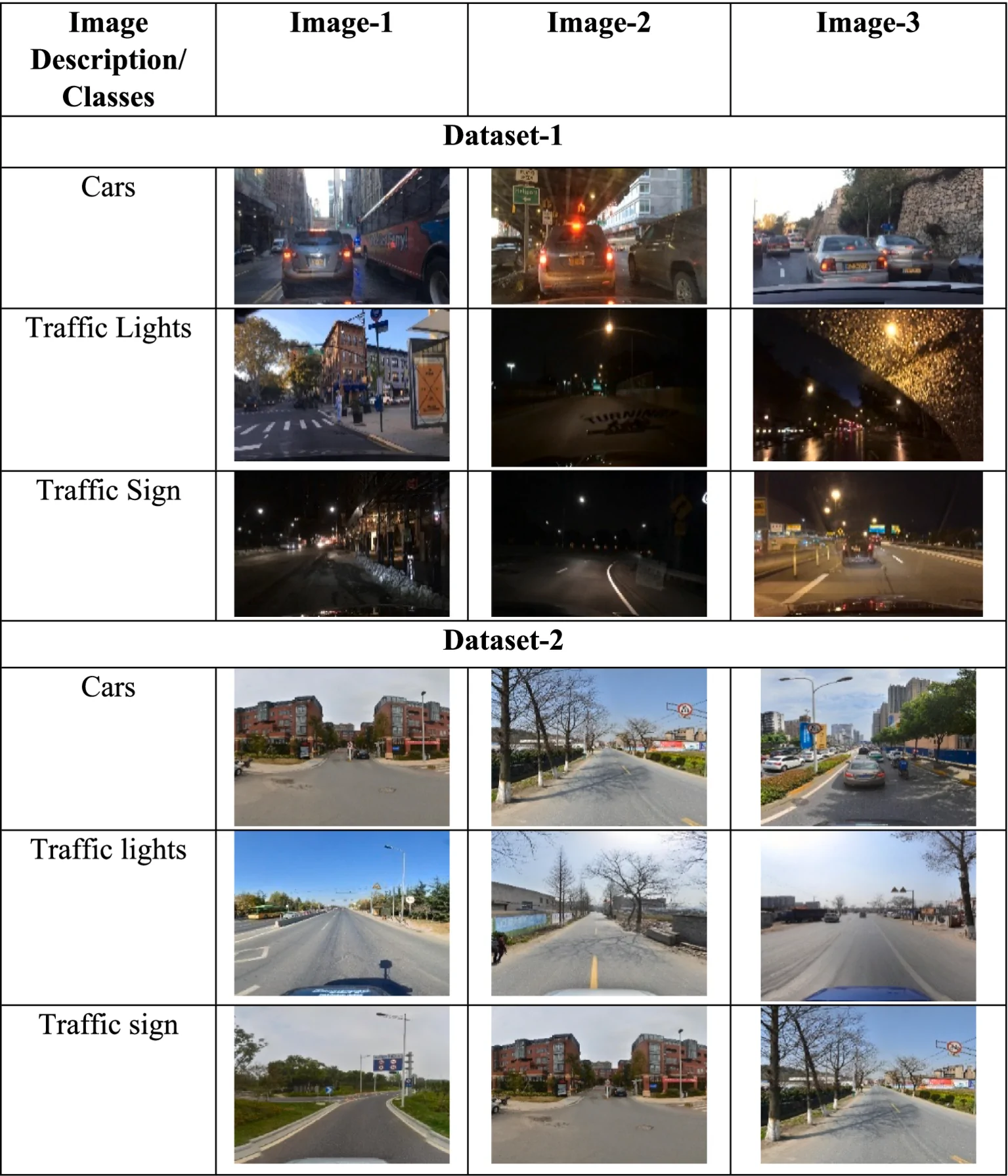
Collected images for the traffic sign detection process (images reproduced with permission from the BDD100K dataset, https://www.kaggle.com/datasets/awsaf49/bdd100k-dataset, licensed under CC0, and the TT100K dataset, https://www.kaggle.com/datasets/braunge/tt100k, licensed under CC-BY 4.0).

*Cross-validation:* Table 7 presents the cross-validation analysis for validating the technique’s robustness. Here, the five-fold cross-validation is conducted on the 2.7% dataset. From the results, the obtained mean accuracy is 96.12 ± 0.19. It illustrates a stable performance in the suggested model across distinct data splits. It also guarantees that the technique is not overfitting to a particular subset. Hence, the utilization of the organized subset is justified for the suggested traffic sign identification process. The dataset details and model training parameters are provided in Tables 8, 9. The collected images are given in Figure 6.

**Table 7:** Cross-validation analysis.

Fold	Accuracy (%)
Fold 1	95.8
Fold 2	96.3
Fold 3	96.0
Fold 4	96.4
Fold 5	96.1
2.7% Mean	96.12 ± 0.18717743453739458
20% Mean	96.6 ± 0.12396128427859872
*P*-value	0.005638983212990391

**Table 8:** Dataset details.

Dataset description							
Dataset	Description	Total Images/ Videos	Used Images / Videos	Reason for data reduction	Training Set (Images)	Test Set (Images)	Image resolution / Size
BDD100K	Large-scale driving video dataset with diverse weather and lighting conditions	100,000 + images	2,763 images	A relevance-based subset is developed to guarantee the balanced class representation and controlled validation of the suggested model.	2072	691	512 × 512 × 3
TT100K	Traffic sign images from real-world urban roads	100,000 + images	3,069 images	A subset is selected to handle the proportional distribution across 49 traffic sign types.	2,302	767	512 × 512 × 3

**Table 9:** Model training parameters.

Component	Parameter	Value
SPP-CAE (Dehazing)	Encoder layers	3 convolution layers
	Kernel sizes	3 × 3
	Activation	ReLU
	Pooling	Max Pooling
	SPP scales	{1 × 1, 2 × 2, 4 × 4}
YOLOv9 Backbone	Input size	512 × 512 × 3
	Anchor boxes	Default YOLOv9
	Loss functions	CIoU + BCE
Training Setup	Epochs	100
	Batch size	32
	Optimizer	Adam
	Initial learning rate	0.001
	LR decay	Cosine
	Momentum	0.937
SAOO-PAS	Population size	10
	Iterations	50
	Chromosome length	3
	Optimized parameters	Learning rate, hidden neurons, activation
	Drone ratio	10%
	Worker ratio	90%

### 6.4 Performance measures

The significant measures used in the model are as follows:

(a) IoU is expressed in Equation 8. In detection validations, the IoU threshold of 0.5 is employed to estimate the true positive detections. It means that a predicted bounding box is represented as a correct detection if its IoU with the ground truth bounding box exceeds 0.5.

(b) Dice coefficient is expressed in Equation 9:


$$\mathrm{DC}=\frac{2|C\cap D|}{|C|+|D|}$$ (9)

(c) Accuracy is expressed in Equation 10:


$$\mathrm{Ac}=\frac{\mathrm{Wp}+\mathrm{Wn}}{\mathrm{Wp}+\mathrm{Wn}+\mathrm{Ip}+\text{In}}$$ (10)

Here, the term Wp is the number of accurately predicted objects, Wn is the number of background pixels correctly predicted as background, Ip is the number of background pixels incorrectly predicted as objects, and In is the number of object pixels incorrectly predicted as background.

### 6.5 Resultant images

**Figure 7:**
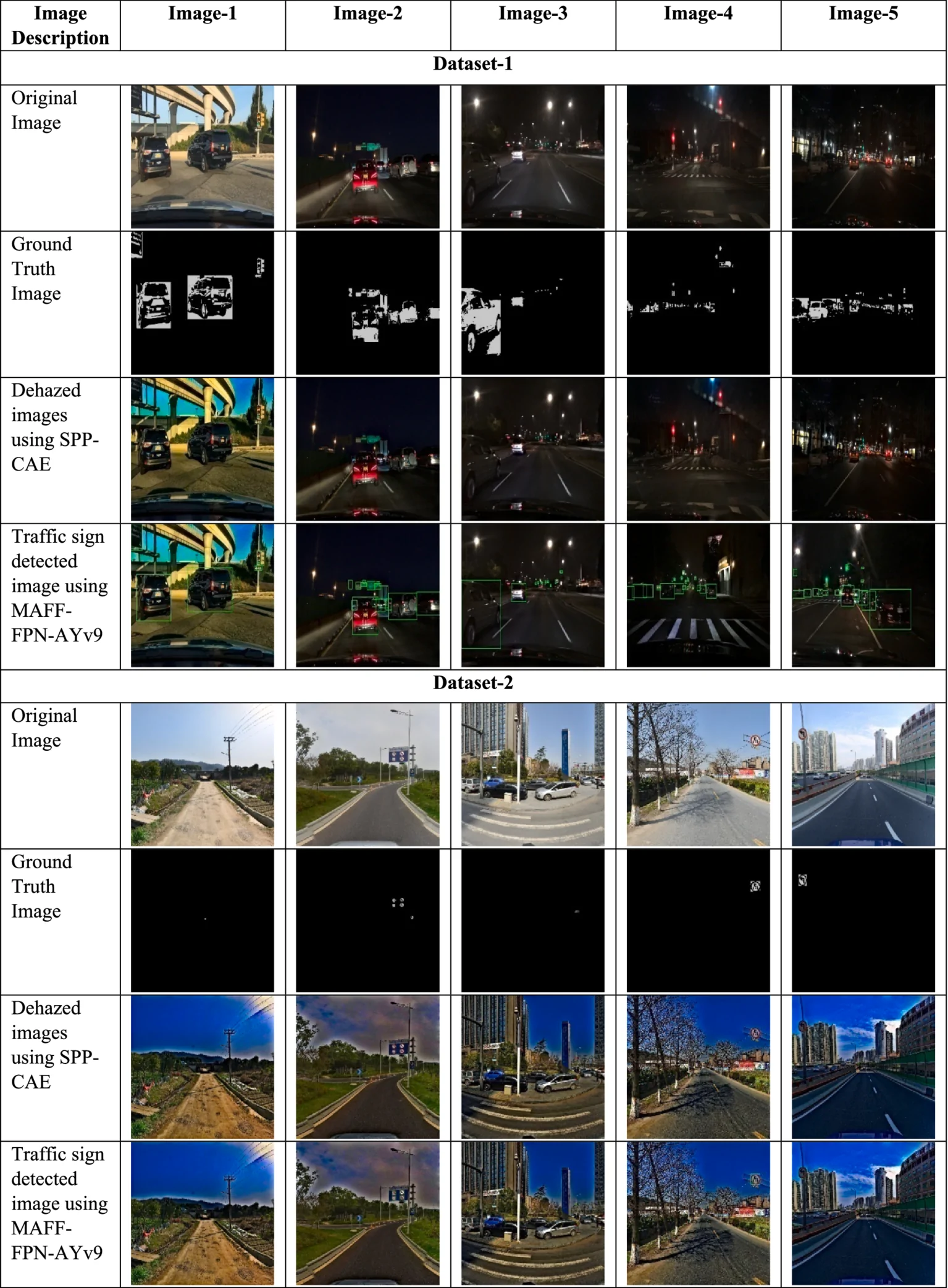
Resultant dehazed images and the traffic sign detected images (images reproduced with permission from the BDD100K dataset, https://www.kaggle.com/datasets/awsaf49/bdd100k-dataset, licensed under CC0, and the TT100K dataset, https://www.kaggle.com/datasets/braunge/tt100k, licensed under CC-BY 4.0).

The resultant dehazed images and the traffic sign detected images from the two benchmark datasets are shown in Figure 7.

### 6.6 Cost function evaluation

**Figure 8:**
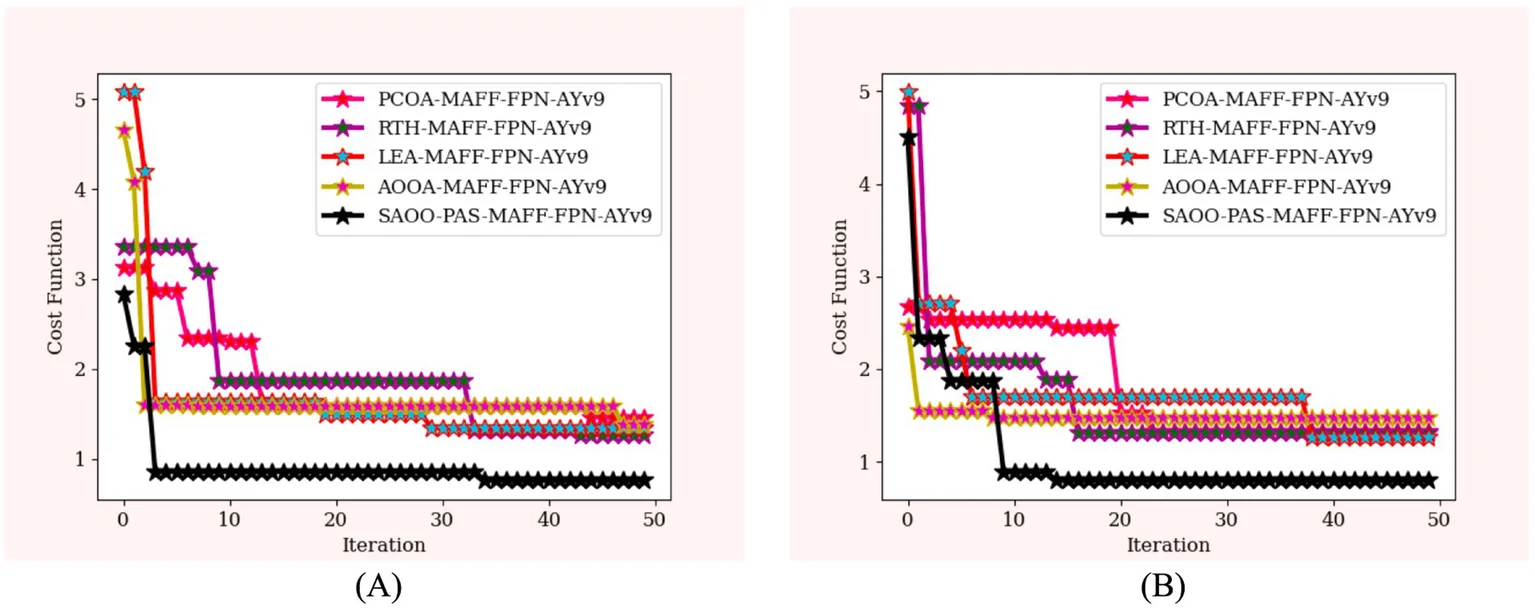
Cost function evaluation of the developed model using **(A)** Dataset 1 and **(B)** Dataset 2.

In this work, the cost function is developed to estimate the overall detection functionality of the designed SAOO-PAS-MAFF-FPN-AYv9 approach. Rather than depending on a single measure, the cost function assists the optimization algorithm toward configurations that improve the robustness and the detection quality. In this suggested work, the cost function is derived based on the maximization of IoU. Since IoU reflects the localization quality, higher IoU values represent better detection accuracy. Hence, the cost function guarantees minimized false positives, quick convergence toward optimal parameter settings, and improved overlap between the ground-truth and predicted bounding boxes. Its experimental validation is provided in Figure 8. The developed traffic sign detection model, which integrates SPP-CAE for image dehazing and MAFF-FPN-AYv9 for traffic sign detection, outperforms several existing models, such as PCOA-MAFF-FPN-AYv9, RTH-MAFF-FPN-AYv9, LEA-MAFF-FPN-AYv9, AOOA-MAFF-FPN-AYv9, and SAOO-PAS-MAFF-FPN-AYv9. The cost function evaluation further confirms that the developed model consistently delivers better results and is effective for intelligent vehicle applications. The developed optimization method provides the most satisfactory solution in the problem domain by avoiding early convergence and local optima issues, which is proven by the convergence results with varying iterations. The optimization performance is greatly improved over prior algorithms.

### 6.7 Traffic sign detection performance evaluation

**Figure 9:**
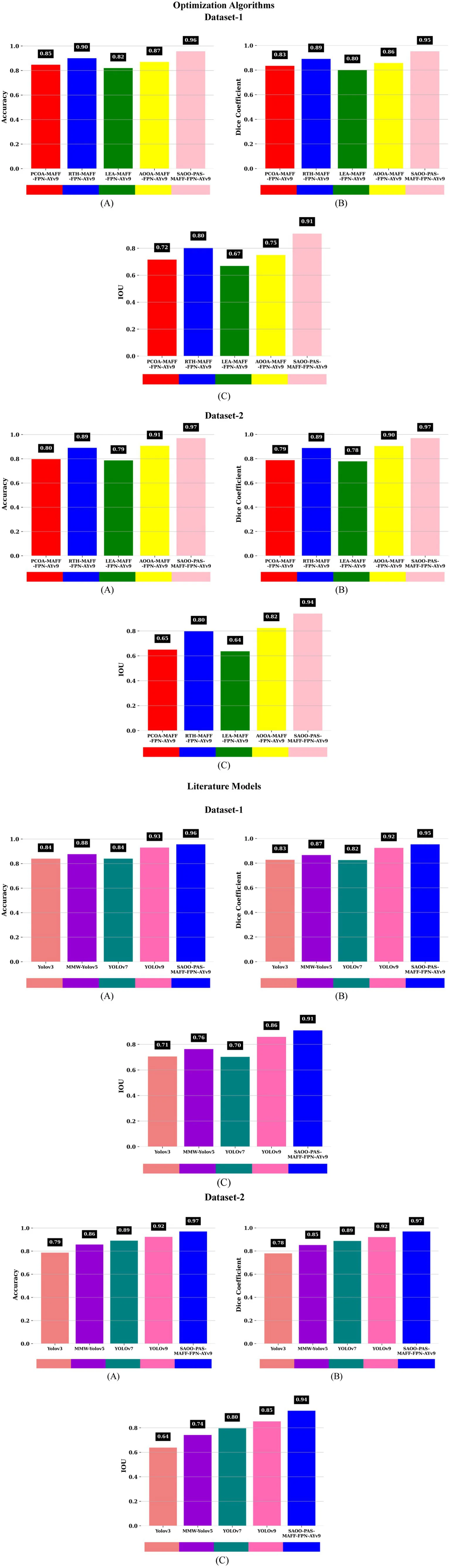
Performance evaluation of the developed model with existing models and optimization algorithms using **(A)** Accuracy, **(B)** Dice coefficient, and **(C)** IoU.

Performance evaluation on the developed traffic sign detection method is given in Tables 10, 11 for different models and optimization algorithms using datasets 1 and 2. It is essential to assess its overall effectiveness and reliability. Here, the accuracy of the developed SAOO-PAS-MAFF-FPN-AYv9 is 96% for dataset-1, and other algorithms such asPCOA-MAFF-FPN-AYv9, RTH-MAFF-FPN-AYv9, LEA-MAFF-FPN-AYv9, and AOOA-MAFF-FPN-AYv9 given the accuracy values to be 85, 90, 82, and 87%. Moreover, the Dice coefficient of the developed SAOO-PAS-MAFF-FPN-AYv9 is 97% for dataset-2, and other algorithms such as PCOA-MAFF-FPN-AYv9, RTH-MAFF-FPN-AYv9, LEA-MAFF-FPN-AYv9, and AOOA-MAFF-FPN-AYv9, give the dice coefficient to be 79, 89, 78, and 90%. Thus, it is proved that the developed traffic sign detection model demonstrates superior efficiency compared to various optimization algorithms and networks. In terms of networks, the developed model also surpasses YOLOv3, MMW-YOLOv5, YOLOv7, and YOLOv9. The developed model ensures faster processing, more precise detection, and increased robustness in varying conditions, making it more reliable in autonomous driving and transportation. The experiment results of the implemented model on BDD100k and TT100K are illustrated in Figure 9.

**Table 10:** Experimental comparison results in the BDD100k dataset.

Optimization Algorithms			
Algorithms	Accuracy	Dice coefficient	IoU
PCOA MAFF-FPN-AYv9	80%	79%	65%
RTH MAFF-FPN-AYv9	89%	89%	80%
LEA MAFF-FPN-AYv9	79%	78%	64%
AOOA MAFF-FPN-AYv9	91%	90%	82%
SAOO-PAS MAFF-FPN-AYv9(proposed)	97%	97%	94%

Literature models			
Models	Accuracy	Dice coefficient	IoU
YOLOv3	79%	78%	64%
MMW YOLOv5	86%	85%	74%
YOLOv7	89%	89%	80%
YOLOv9	92%	92%	85%
SAOO-PAS MAFF-FPN-AYv9(proposed)	97%	97%	94%

**Table 11:** Experimental comparison results in the TT100k dataset.

Optimization algorithms			
Algorithms	Accuracy	Dice Coefficient	IoU
PCOA MAFF-FPN-AYv9	85%	83%	72%
RTH MAFF-FPN-AYv9	90%	89%	80%
LEA MAFF-FPN-AYv9	82%	80%	67%
AOOA MAFF-FPN-AYv9	87%	86%	75%
SAOO-PAS MAFF-FPN-AYv9(proposed)	96%	95%	91%

Literature models			
Models	Accuracy	Dice coefficient	IoU
YOLOv3	84%	83%	71%
MMW YOLOv5	88%	87%	76%
YOLOv7	84%	82%	70%
YOLOv9	93%	92%	86%
SAOO-PAS MAFF-FPN-AYv9(proposed)	96%	95%	91%

### 6.8 Comparative evaluation of the developed model

A comparative evaluation of the developed traffic sign detection model is given in Table 12. The performance is compared with several optimization algorithms, including PCOA-MAFF-FPN-AYv9, RTH-MAFF-FPN-AYv9, LEA-MAFF-FPN-AYv9, AOOA-MAFF-FPN-AYv9, and SAOO-PAS-MAFF-FPN-AYv9, using the dice coefficient as a metric. The Dice coefficient values achieved by each algorithm are as follows: PCOA-MAFF-FPN-AYv9 scored 88.822, RTH-MAFF-FPN-AYv9 achieved 89.395, LEA-MAFF-FPN-AYv9 recorded 90.750, AOOA-MAFF-FPN-AYv9 reached 91.183, and SAOO-PAS-MAFF-FPN-AYv9 showed the highest performance with a dice coefficient of 95.639 when evaluating the best score. These results indicate that the SAOO-PAS-MAFF-FPN-AYv9 significantly outperforms the others in terms of segmentation accuracy, demonstrating its superior ability to detect traffic signs with higher precision and consistency. Furthermore, in comparison to networks like YOLOv3, MMW-YOLOv5, YOLOv7, YOLOv9, and SAOO-PAS-MAFF-FPN-AYv9, the developed model outperforms these existing approaches in terms of detection precision, multi-scale feature handling, and overall reliability.

**Table 12:** Comparative evaluation of the developed model.

Terms	PCOA-MAFF-FPN-AYv9 (Valikhan Anaraki and Farzin, 2024)	RTH-MAFF-FPN-AYv9 (Ferahtia et al., 2023)	LEA-MAFF-FPN-AYv9 (Dalirinia et al., 2024)	AOOA-MAFF-FPN-AYv9 (Al-Sharqi et al., 2024)	SAOO-PAS-MAFF-FPN-AYv9
Dataset-1					
Dice coefficient					
BEST	88.822	89.395	90.750	91.183	95.639
WORST	88.643	85.046	85.293	86.754	92.581
MEAN	88.697	87.435	88.300	88.960	94.380
MEDIAN	88.680	87.661	88.188	88.325	94.255
STD	0.051	1.517	1.598	1.631	0.947
Jaccard coefficient					
BEST	79.892	80.824	83.067	83.795	91.643
WORST	79.603	73.982	74.357	76.606	86.187
MEAN	79.689	77.707	79.088	80.155	89.373
MEDIAN	79.663	78.032	78.872	79.091	89.135
STD	0.082	2.390	2.554	2.653	1.696
Accuracy					
BEST	88.725	89.491	90.582	90.414	95.395
WORST	88.660	84.970	85.190	86.539	92.566
MEAN	88.682	87.360	88.094	88.694	94.234
MEDIAN	88.673	87.289	88.152	88.252	94.073
STD	0.022	1.509	1.578	1.360	0.843
Dataset-2					
Dice coefficient					
BEST	88.786	88.508	89.784	91.182	95.222
WORST	88.588	84.932	86.143	86.393	93.471
MEAN	88.695	86.943	88.112	88.668	94.368
MEDIAN	88.706	87.157	88.345	88.060	94.416
STD	0.063	1.066	1.407	1.825	0.539
Jaccard coefficient					
BEST	79.833	79.385	81.462	83.792	90.881
WORST	79.514	73.810	75.659	76.046	87.742
MEAN	79.687	76.917	78.779	79.692	89.341
MEDIAN	79.705	77.238	79.132	78.673	89.422
STD	0.101	1.660	2.242	2.957	0.964
Accuracy					
BEST	88.771	88.509	89.877	90.788	95.203
WORST	88.651	84.790	85.847	86.333	93.340
MEAN	88.697	86.900	87.999	88.544	94.305
MEDIAN	88.686	87.121	88.191	87.963	94.308
STD	0.043	1.081	1.333	1.676	0.555


Terms	YOLOv3 (Xiong et al., 2023)	MMW-YOLOv5 (Wang T. et al., 2024)	YOLOv7 (Kuppusamy et al., 2023)	YOLOv9 (Rizzieri et al., 2024)	SAOO-PAS-MAFF-FPN-AYv9
Dataset-1					
Dice coefficient					
BEST	89.231	89.143	88.903	89.555	95.639
WORST	82.870	85.413	84.068	86.745	92.581
MEAN	86.234	87.401	86.981	88.075	94.380
MEDIAN	86.636	87.105	87.556	87.832	94.255
STD	1.946	1.045	1.592	1.000	0.947
Jaccard coefficient					
BEST	80.557	80.412	80.023	81.086	91.643
WORST	70.751	74.541	72.515	76.593	86.187
MEAN	75.850	77.637	76.996	78.705	89.373
MEDIAN	76.428	77.155	77.866	78.304	89.135
STD	3.001	1.651	2.469	1.599	1.696
Accuracy					
BEST	88.657	88.800	88.622	89.792	95.395
WORST	82.785	85.373	83.789	86.107	92.566
MEAN	85.892	87.127	86.857	88.012	94.234
BEST	85.986	87.263	87.463	87.874	94.073
WORST	1.797	1.043	1.594	1.116	0.843
Dataset-2					
Dice coefficient					
BEST	88.408	89.647	89.471	89.947	95.222
WORST	84.512	85.379	86.487	86.887	93.471
MEAN	86.528	87.497	88.082	88.169	94.368
MEDIAN	86.610	88.126	88.156	88.295	94.416
STD	1.228	1.419	0.942	0.901	0.539
Jaccard coefficient					
BEST	79.224	81.236	80.947	81.730	90.881
WORST	73.179	74.488	76.191	76.815	87.742
MEAN	76.276	77.801	78.715	78.853	89.341
MEDIAN	76.384	78.772	78.822	79.044	89.422
STD	1.907	2.237	1.501	1.445	0.964
Accuracy					
BEST	88.229	89.348	89.479	89.600	95.203
WORST	84.753	85.468	86.089	87.032	93.340
MEAN	86.415	87.375	87.934	88.081	94.305
MEDIAN	86.369	87.270	88.240	87.943	94.308
STD	1.140	1.223	0.903	0.638	0.555

### 6.9 Statistical evaluation of the proposed model

Table 13 displays a statistical analysis of the proposed model. The developed model’s performance is assessed by some of the measures, such as best, worst, mean, median, and standard deviation. Here, the existing optimization algorithms, like PCOA-MAFF-FPN-AYv9, RTH-MAFF-FPN-AYv9, LEA-MAFF-FPN-AYv9, and AOOA-MAFF-FPN-AYv9, are used for comparison with the proposed SAOO-PAS-MAFF-FPN-AYv9. When evaluating the performance, the developed SAOO-PAS-MAFF-FPN-AYv9 attained the best value of 0.759, proving its enhanced ability over other optimization algorithms. PCOA-MAFF-FPN-AYv9 recorded 1.464, RTH-MAFF-FPN-AYv9 achieved 1.268, LEA-MAFF-FPN-AYv9 scored 1.342, and AOOA-MAFF-FPN-AYv9 attained 1.383. This indicates that the SAOO-PAS-MAFF-FPN-AYv9 optimization algorithm demonstrates superior effectiveness, delivering more precise results compared to the other optimization algorithms tested on Dataset-1.

**Table 13:** Statistical evaluation of the proposed model.

Terms	PCOA-MAFF-FPN-AYv9	RTH-MAFF-FPN-AYv9	LEA-MAFF-FPN-AYv9	AOOA-MAFF-FPN-AYv9	SAOO-PAS-MAFF-FPN-AYv9
Dataset-1					
BEST	1.464	1.268	1.342	1.383	0.759
WORST	3.133	3.358	5.074	4.655	2.834
MEDIAN	1.584	1.863	1.502	1.581	0.858
MEAN	1.845	1.928	1.676	1.682	0.922
STD	0.505	0.693	0.801	0.553	0.394
Dataset-2					
BEST	1.330	1.311	1.261	1.480	0.798
WORST	2.678	4.838	4.986	2.461	2.236
MEDIAN	1.330	1.319	1.705	1.480	0.798
MEAN	1.819	1.661	1.755	1.509	1.082
STD	0.579	0.727	0.588	0.138	0.674

### 6.10 Diversity evaluation of developed model

Table 14 gives the diversity of the BDD100K and TT100K data sources. The BDD100K data source includes over 100,000 images garnered from highway and urban driving in the USA. It considers distinct weather, traffic complexity, and lighting conditions with 1,280 × 720p resolution. On the other hand, the TT100K dataset includes 100,000 images with 2048 × 2048p resolution. This data source includes more than 220 traffic sign classes from Chinese roads. Although these statistics explain the dataset characteristics, they also give a benchmark for estimating the robustness of the technique. The designed MAFF-FPN-AYv9 technique illustrates high detection functionality across both data sources. Especially, the designed MAFF-FPN-AYv9 attains strong IoU, dice coefficient, and mAP@0.5 values even for rare traffic sign classes. This finding indicates that the technique is capable of identifying traffic signs effectively under dynamic environmental conditions. Thus, the designed MAFF-FPN-AYv9 ensures its robustness for real-world autonomous driving scenarios.

**Table 14:** Diversity evaluation on benchmark datasets.

Dataset	Domain	Total Images / Videos	Image Resolution	Diversity & Challenges	Usefulness
BDD100K	Driving (USA, urban and highway)	100,000 + images	1,280 × 720p	Large-scale, including weather, lighting variations, and traffic complexity	Strong benchmark for real-world autonomous driving
TT100K	Traffic signs (China)	100,000 + images	2048 × 2048p	>220 classes of signs, unbalanced distribution, varied backgrounds	Good for fine-grained traffic sign detection

### 6.11 Performance comparison over desmogging techniques

Table 15 provides the traffic sign detection performance of the suggested method with the traditional desmogging techniques. Previously developed various desmogging techniques are considered for the evaluation of performance in terms of the measures such as detection accuracy, perceptual metric, and run time. The measures such as Peak Signal to Noise Ratio (PSNR), Structural Similarity Index Measure (SSIM) and Natural Image Quality Estimator (NIQE↓) are used to ensure the performance. The higher PSNR values indicate a higher quality of the image, which means that there is less error. SSIM is a perceptual measure for evaluating the quality of images based on the structural changes. NIQE↓ indicates the natural quality of the model, and the lower the NIQE score, indicates that the image is more natural. The results clearly show that the detection accuracy of our proposed approach is 96%, which is more impressive than the previous desmogging techniques such as Dark Channel Prior (DCP), Non-local Image Dehazing (NLD), All-in-One Network for Dehazing (AOD-Net), and Gated Context Aggregation Network (GCANet). The residual regression network with morphological erosion attained more effective traffic sign detection results than the previous methods. But our developed model attains higher traffic sign detection accuracy with reduced run time than the Residual regression network (RRNet)-Morphological erosion method. In addition, the suggested work leverages the SPP-CAE for the image dehazing operation. The designed SPP-CAE dehazing approach works as a lightweight AE model. It is processed once per input frame before identifying the objects. Since it is fully conventional and does not perform iterative refinement, the SPP-CAE model’s computational overhead is low. Therefore, the run time of the suggested image becomes much lower than that of the traditional models.

**Table 15:** Performance comparison over desmogging techniques.

Method	PSNR (dB)	SSIM	Perceptual Metric (NIQE↓)	Detection Accuracy (mAP%)	Run time (sec/image)
DCP (He et al. 2011; Juneja et al., 2023c)	17.8	0.72	High (poor quality)	84.1	0.35
NLD (Berman et al. 2016; Juneja et al., 2023c)	19.2	0.75	Medium	86.4	0.40
AOD-Net (Li et al. 2017; Juneja et al., 2023c)	21.5	0.8	Medium	89.2	0.20
GCANet (Chen et al. 2019; Juneja et al., 2023c)	22.7	0.83	Lower	90.5	0.25
RRNet + Morph. Erosion (Juneja et al., 2023a; Juneja et al., 2023b; Juneja et al., 2023c; Juneja et al., 2024)	24.8	0.88	Low	93.6	0.45
SPP-CAE + MAFF-FPN-AYv9 + SAOO-PAS (proposed)	25.6	0.90	Lowest	96.0	0.28

### 6.12 5-fold cross-validation performance across datasets

**Figure 10:**
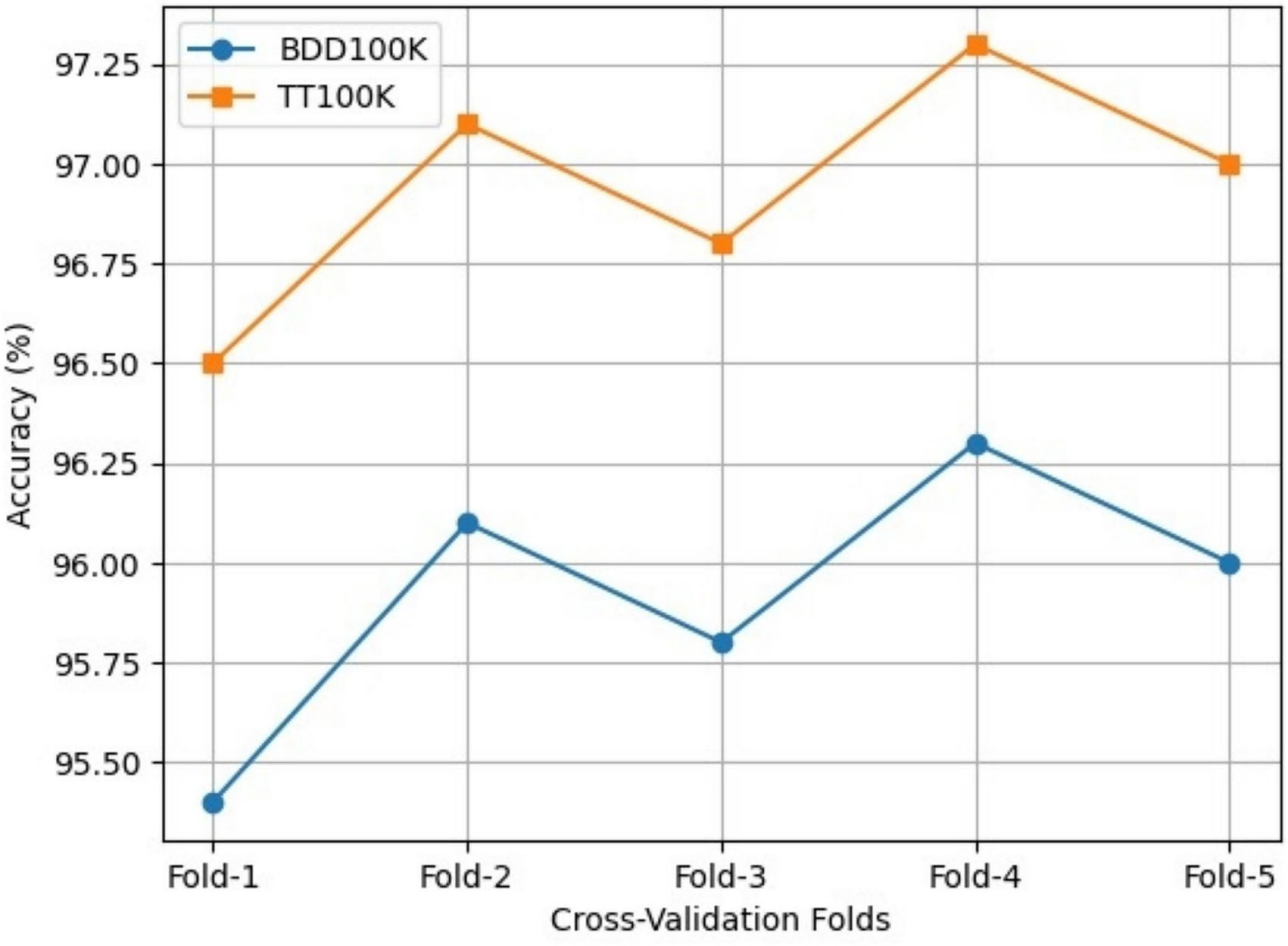
Five-fold cross-validation performance across datasets.

Figure 10 gives the 5-fold cross-validation performance across datasets. The suggested work leverages two standard datasets for the validation, and their cross-validation is shown here. To guarantee the generalization capacity and the robustness of the designed model, a 5-fold cross-validation mechanism is utilized. The results elucidated that the designed model attains high detection accuracy with very low variance. It demonstrates the high adaptability and robustness of the designed framework across distinct traffic sign data sources. In addition, the results guarantee the suitability of the designed approach in smart transport systems working under complex environmental conditions.

### 6.13 Comparative analysis with recent SOTA methods

Table 16 gives the comparative validation of the implemented SAOO-PAS-MAFF-FPN-AYv9 with recent SOTA approaches. This investigation illustrates the efficiency of the designed SAOO-PAS-MAFF-FPN-AYv9. Contrasted to the SOTA techniques such as DLHR-TSDR and TSD-DETR, the designed SAOO-PAS-MAFF-FPN-AYv9 attains higher detection accuracy (95%). The sensitivity and specificity rates of the designed SAOO-PAS-MAFF-FPN-AYv9 are also high for the suggested model in both datasets. The inclusion of the MAFF improves the feature representation across scales. It results in promising performance rates. In addition, the SAOO-PAS-aided parameter optimization supports highly effective convergence and better performance than other optimization mechanisms. From these results, it is ensured that the recommended SAOO-PAS-MAFF-FPN-AYv9 outperforms the existing SOTA approaches. Also, the designed SAOO-PAS-MAFF-FPN-AYv9 reduces the false positives more than the recent SOTA methods.

**Table 16:** Comparative analysis with recent SOTA models.

Terms	DLHR-TSDR (Rani et al., 2024)	TSD-DETR (Zhang L. et al., 2025)	Lightweight vehicle-mounted multi-scale TSD (Wang et al. (2024a))	Autoencoder-Resnet-101 (Juneja et al. (2023a))	SAOO-PAS-MAFF-FPN-AYv9
Dataset-1					
Dice coefficient	0.927	0.929	0.932	0.925	0.955
Jaccard	0.864	0.868	0.873	0.862	0.914
Accuracy	0.930	0.929	0.930	0.926	0.952
Sensitivity	0.922	0.930	0.942	0.923	0.953
Specificity	0.937	0.927	0.918	0.929	0.950
Precision	0.931	0.927	0.923	0.927	0.957
FPR	0.062	0.072	0.081	0.070	0.049
FNR	0.077	0.069	0.057	0.076	0.046
NPV	0.937	0.927	0.918	0.929	0.950
FDR	0.068	0.072	0.076	0.072	0.042
F1-Score	0.927	0.929	0.932	0.925	0.955
MCC	0.861	0.858	0.861	0.852	0.903
Dataset-2					
Dice coefficient	0.939	0.918	0.940	0.931	0.944
Jaccard	0.885	0.849	0.887	0.872	0.894
Accuracy	0.940	0.921	0.940	0.932	0.941
Sensitivity	0.950	0.917	0.947	0.950	0.945
Specificity	0.930	0.924	0.932	0.917	0.938
Precision	0.928	0.919	0.933	0.913	0.943
FPR	0.069	0.075	0.067	0.082	0.061
FNR	0.049	0.082	0.052	0.049	0.054
NPV	0.930	0.924	0.932	0.917	0.938
FDR	0.071	0.080	0.066	0.086	0.056
F1-Score	0.939	0.918	0.940	0.931	0.944
MCC	0.880	0.842	0.880	0.866	0.883

### 6.14 Analysis of quantitative dehazing performance

**Figure 11:**
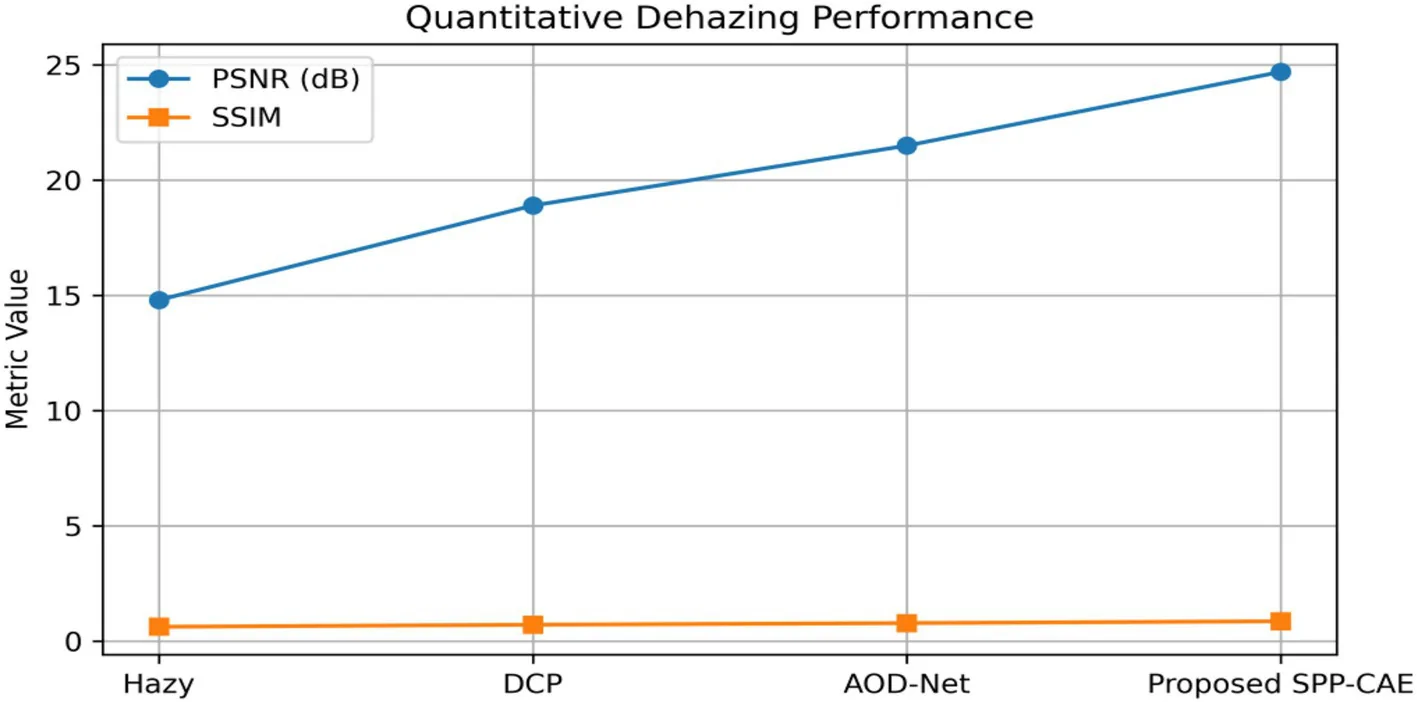
Analysis of quantitative dehazing performance.

Figure 11 provides the quantitative dehazing performance validation of the designed SPP-CAE. The quantitative efficiency of the designed SPP-CAE-aided image dehazing process is estimated by employing the well-known image quality validation measures to estimate the improvement attained during preprocessing. The measures PSNR and SSIM are utilized to validate the noise minimization and restoration quality. The quantitative dehazing outcomes ensured that the designed SPP-CAE mechanism highly improves the quality of an image and plays a significant part in improving the accuracy and robustness of the traffic sign detection approach under complex scenarios.

### 6.15 Quantitative experimental results of developed MAFF-FPN

Table 17 presents the quantitative experimental results of the developed MAFF-FPN in the suggested work. The findings elaborate on the efficiency of the designed MAFF-FPN over the existing FPN. Including the attention-based multi-scale feature fusion improves the recall and precision by over 3%. It illustrates that highly accurate and consistent outcomes are achieved by the designed MAFF-FPN. The dice and IoU measures show superior localization, most importantly for the tiny traffic signs. Hence, the experiments elucidate that the designed MAFF-FPN is better than the traditional FPN models, and it is also highly suitable for the proposed work.

**Table 17:** Quantitative experimental results of developing MAFF-FPN.

Model	Precision (%)	Recall (%)	IoU (%)	Dice (%)	mAP@0.5 (%)
YOLOv9 + FPN	94.12	92.88	89.47	90.21	95.06
YOLOv9 + MAFF-FPN (proposed)	97.35	96.11	93.52	94.18	98.27

### 6.16 Per-class performance analysis

**Figure 12:**
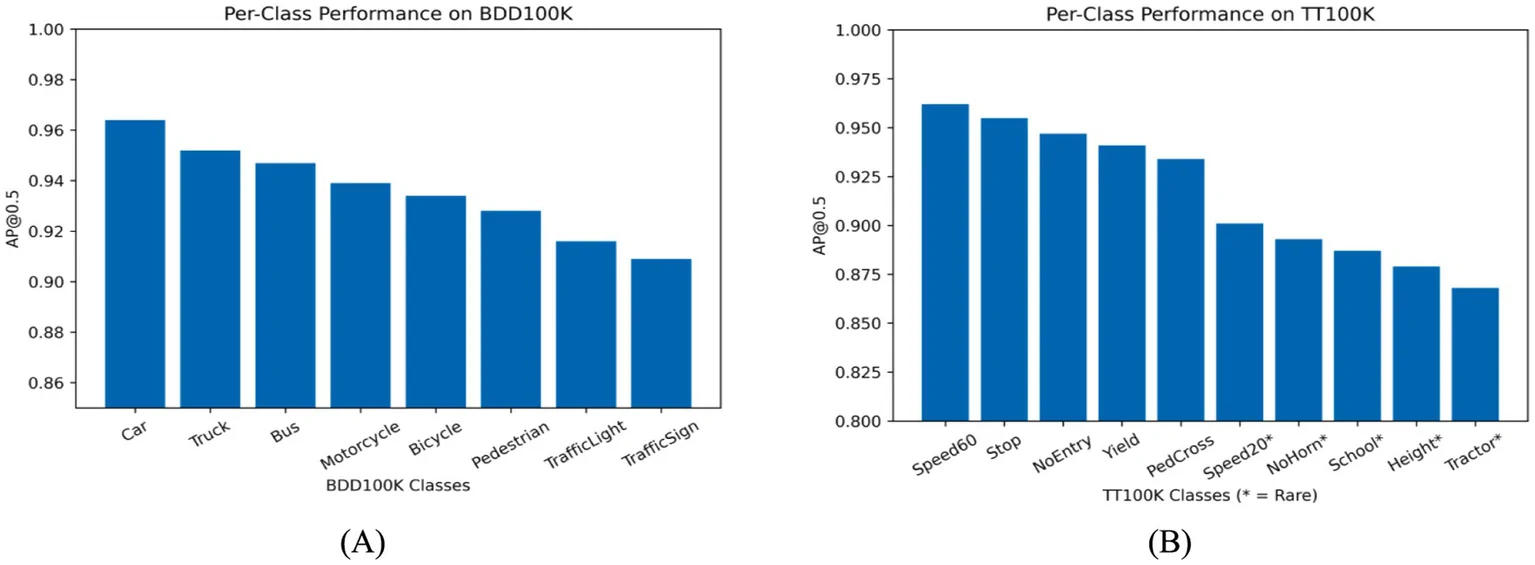
Per-class performance analysis in terms of **(A)** BDD100k and **(B)** TT100k.

Figure 12 shows the per-class performance of the suggested approach. For two data sources, this validation is conducted. Per-class performance defines validating the technique’s detection accuracy individually for each class. This analysis provided detailed validation of technique robustness and class-specific behavior beyond overall accuracy. In addition, Table 18 provides the per-class performance detection on the two standard datasets. The per-class analysis guarantees that the suggested technique does not extremely favor the dominant types and handles stable detection functionality across both rate and often traffic signs.

**Table 18:** Per-class performance detection on two datasets.

BDD100K				
Class	#Instances	AP@0.5	Precision	Recall
Car	18,540	0.964	0.97	0.958
Truck	4,320	0.952	0.958	0.944
Bus	3,110	0.947	0.953	0.939
Motorcycle	2,870	0.939	0.944	0.932
Bicycle	2,410	0.934	0.938	0.928
Pedestrian	5,230	0.928	0.933	0.921
Traffic Light	1,280	0.916	0.922	0.907
Traffic Sign	1,140	0.909	0.914	0.902

TT100K				
Class	#Instances	AP@0.5	Precision	Recall
Speed Limit 60	1,450	0.962	0.968	0.955
Stop	1,320	0.955	0.96	0.948
No Entry	1,187	0.947	0.952	0.939
Yield	1,035	0.941	0.945	0.936
Pedestrian Crossing	865	0.934	0.938	0.928
Speed Limit 20 (Rare)	92	0.901	0.912	0.884
No Horn (Rare)	76	0.893	0.904	0.876
School Zone (Rare)	64	0.887	0.895	0.872
Height Limit (Rare)	58	0.879	0.886	0.861
Tractor Warning (Rare)	41	0.868	0.874	0.852

### 6.17 Data augmentation effects

**Figure 13:**
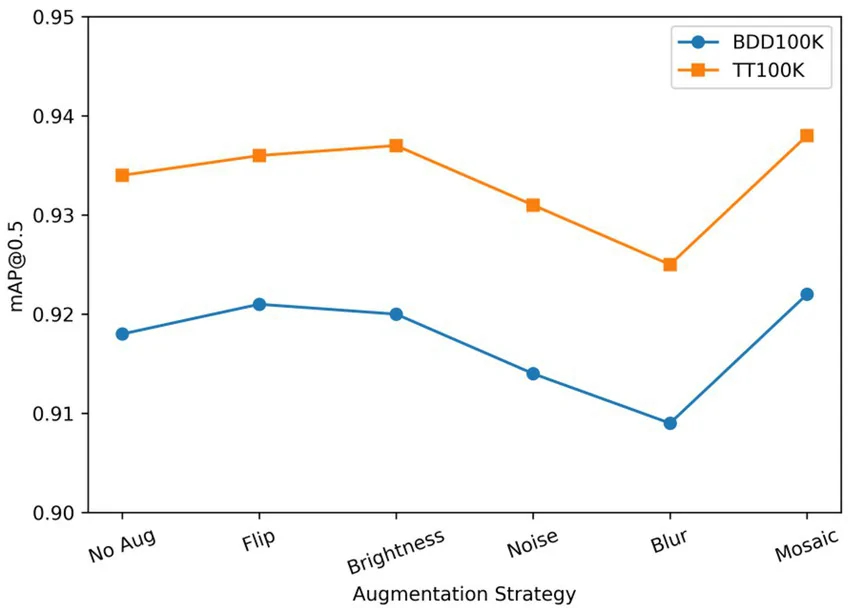
Effect of data augmentation on two standard datasets.

The suggested work performs data augmentation tasks by considering the operations, such as horizontal flip, random brightness, Gaussian noise, random blur, and mosaic. These operations improve the functionality of the designed technique by artificially increasing the dataset diversity and size. The effects of data augmentation on two data sources are provided in Figure 13. Both data sources show better improvements in the data augmentation process. To estimate the efficiency of the data augmentation, a study is performed for both datasets, and is shown in Table 19. For the BDD100k subset (2.7% of the full dataset) and for the TT100k dataset, the augmentation gives minor enhancements in identification functionality. Thus, these results elucidate that the carefully chosen subset captures enough variability. Though the outcomes offer tiny enhancements, the gains are constrained. For the BDD100k 2.7% subset, the image selection guaranteed enough diversity in the traffic conditions, lighting, and weather conditions. Hence, the extra augmented samples only offer average merit, and the main validation of the suggested approach utilizing original images is enough to indicate its original functionality.

**Table 19:** Impact of data augmentation on two datasets.

Configuration	BDD100K		TT100K	
	mAP@0.5	Δ vs No Aug	mAP@0.5	Δ vs No Aug
No augmentation	0.918	–	0.934	–
Flip	0.921	+0.3%	0.936	+0.2%
Brightness	0.920	+0.2%	0.937	+0.3%
Gaussian noise	0.914	−0.4%	0.931	−0.3%
Blur	0.909	−0.9%	0.925	−0.9%
Mosaic	0.922	+0.4%	0.938	+0.4%

### 6.18 Ablation study

To validate the contribution of each component in the suggested model, an ablation study is performed on both the BDD100K and TT100K data sources. The results are given in Table 20. When considering the BDD100K data source, the YOLOv9 + MAFF-FPN method attains a Dice coefficient of 0.849. Including the SPP-CAE dehazing block highly enhances the performance to 0.907. It represents that the image restoration operation plays a significant part in improving the feature separability under critical weather conditions. Nevertheless, when the dehazing block is eliminated, the performance degrades to 0.825 in the dice coefficient. It guarantees that the visibility improvement is a primary factor in adverse conditions. The inclusion of the SAOO-PAS-aided optimization process further enhances the performance to a 0.952 dice coefficient. It illustrates that the effective hyperparameter optimization supports highly stable convergence and enhanced feature learning efficiency. The suggested Dehazing + MAFF-FPN + SPP-CAE + SAOO-PAS attained the best performance across all performance measures. It highlights the total gain of the designed approach in discriminative capacity. When observing the TT100K dataset, similar improvements are noted. It is guaranteed that the enhancements generalize across the data sources with distinct class distributions. Thus, the results illustrate that the designed Dehazing + MAFF-FPN + SPP-CAE + SAOO-PAS improves traffic sign detection efficiently.

**Table 20:** Ablation study on two datasets.

BDD100K				
Terms	YOLOv9 + MAFF-FPN	YOLOv9 + MAFF-FPN + SPP-CAE	YOLOv9 + MAFF-FPN + SPP-CAE + SAOO-PAS (No Dehazing)	Dehazing + MAFF-FPN + SPP-CAE + SAOO-PAS (Proposed)
Dice coefficient	0.849	0.907	0.825	0.952
Jaccard	0.738	0.830	0.703	0.908
Accuracy	0.843	0.903	0.820	0.950
Sensitivity	0.847	0.904	0.815	0.949
Specificity	0.839	0.902	0.825	0.951
Precision	0.852	0.910	0.836	0.955
FPR	0.160	0.097	0.174	0.048
FNR	0.152	0.095	0.184	0.050
NPV	0.839	0.902	0.825	0.951
FDR	0.147	0.089	0.163	0.044
F1-Score	0.849	0.907	0.825	0.952
MCC	0.686	0.806	0.639	0.899

TT100K				
Terms	YOLOv9 + MAFF-FPN	YOLOv9 + MAFF-FPN + SPP-CAE	YOLOv9 + MAFF-FPN + SPP-CAE + SAOO-PAS (No Dehazing)	Dehazing + MAFF-FPN + SPP-CAE + SAOO-PAS (Proposed)
Dice Coefficien	0.938	0.890	0.883	0.941
Jaccard	0.884	0.802	0.791	0.889
Accuracy	0.936	0.886	0.880	0.940
Sensitivity	0.935	0.890	0.883	0.935
Specificity	0.937	0.882	0.875	0.944
Precision	0.941	0.890	0.883	0.947
FPR	0.062	0.117	0.124	0.055
FNR	0.064	0.109	0.116	0.064
NPV	0.937	0.882	0.875	0.944
FDR	0.058	0.109	0.116	0.052
F1-Score	0.938	0.890	0.883	0.941
MCC	0.873	0.773	0.759	0.879

### 6.19 Statistical significance test

Table 21 provides the statistical significance test for the suggested model. To estimate the performance enhancements of the designed SAOO-PAS-MAFF-FPN-AYv9 technique, a statistical significance test is carried out. In this analysis, the *t*-tests are paired to contrast the suggested SAOO-PAS-MAFF-FPN-AYv9 with existing methods. In addition, the Friedman Aligned Ranks test is performed for distinct model estimation. The null hypothesis elucidates that there is no significant change in the identification functionality between the baseline and suggested methods. The statistical test shows the mean performance change over distinct runs, while the *p*-value shows the probability of monitoring the estimated enhancement under H_0_. The comparison displays *p*-values < 0.005, narrow confidence intervals, and high effect sizes. It elucidates the statistically significant enhancements of the suggested framework over the existing methods.

**Table 21:** Statistical significance test.

SAOO-PAS optimization across YOLO techniques					
Comparison	Mean gain	*p*-value	95% CI	Cohen’s *d*	Significant?
YOLOv9 vs. SAOO-PAS	3.00%	<0.0001	[2.8, 3.2%]	15.4	Yes
YOLOv7 vs. SAOO-PAS	2.90%	0.0002	[2.5, 3.3%]	8.9	Yes
YOLOv5 vs. SAOO-PAS	3.30%	0.0001	[2.9, 3.7%]	9.4	Yes

Friedman Aligned Ranks test (significance level of 0.005)			
Comparison	Statistic	Adjusted *p*-value	Result
PCOA-MAFF-FPN-AYv9 (Valikhan Anaraki and Farzin, 2024) vs. SAOO-PAS-MAFF-FPN-AYv9	1.788	0.736	H_0_ is accepted
LEA-MAFF-FPN-AYv9 (Dalirinia et al., 2024) vs. PCOA-MAFF-FPN-AYv9 (Valikhan Anaraki and Farzin, 2024)	1.341	1	
RTH-MAFF-FPN-AYv9 (Ferahtia et al., 2023) vs. SAOO-PAS-MAFF-FPN-AYv9	1.341	1	
LEA-MAFF-FPN-AYv9 (Dalirinia et al., 2024) vs. RTH-MAFF-FPN-AYv9 (Ferahtia et al., 2023)	0.894	1	
PCOA-MAFF-FPN-AYv9 (Valikhan Anaraki and Farzin, 2024) vs. AOOA-MAFF-FPN-AYv9 (Al-Sharqi et al., 2024)	0.894	1	
SAOO-PAS-MAFF-FPN-AYv9 vs. AOOA-MAFF-FPN-AYv9 (Al-Sharqi et al., 2024)	0.894	1	
LEA-MAFF-FPN-AYv9 (Dalirinia et al., 2024) vs. SAOO-PAS-MAFF-FPN-AYv9	0.447	1	
RTH-MAFF-FPN-AYv9 (Ferahtia et al., 2023) vs. PCOA-MAFF-FPN-AYv9 (Valikhan Anaraki and Farzin, 2024)	0.447	1	
RTH-MAFF-FPN-AYv9 (Ferahtia et al., 2023) vs. AOOA-MAFF-FPN-AYv9 (Al-Sharqi et al., 2024)	0.447	1	
LEA-MAFF-FPN-AYv9 (Dalirinia et al., 2024) vs. AOOA-MAFF-FPN-AYv9 (Al-Sharqi et al., 2024)	0.447	1	

### 6.20 Generalization of proposed dehazing + SAOO-PAS across YOLO variants

**Figure 14:**
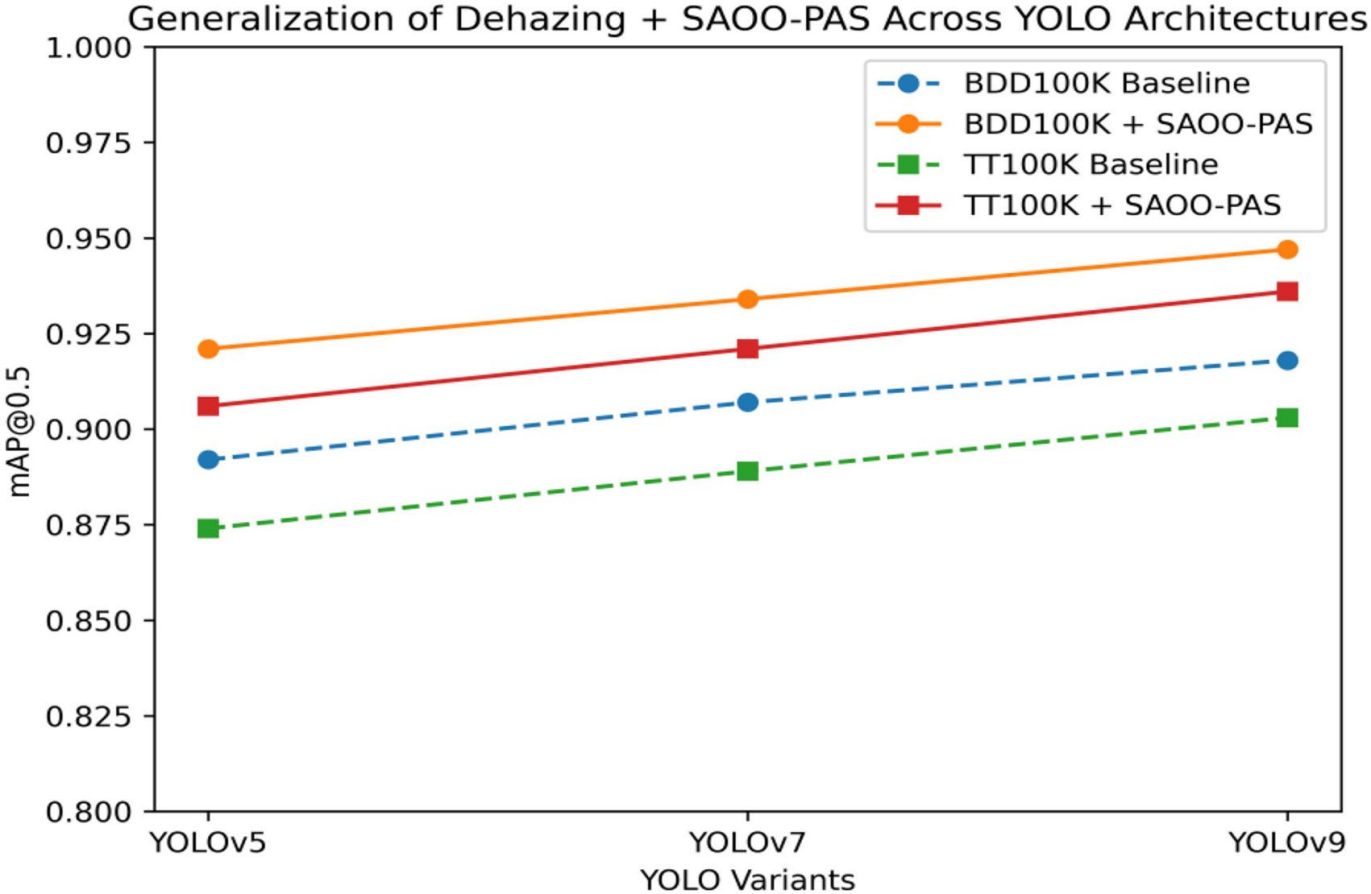
Generalization of proposed dehazing + SAOO-PAS across YOLO variants.

Figure 14 depicts the efficiency and generalizability of the designed dehazing task integrated with the SAOO-PAS optimization across distinct YOLO variants, such as YOLOv5, YOLOv7, and YOLOv9, on two data sources. The dashed lines specify the baseline techniques, and the solid lines represent performance after employing the dehazing and SAOO-PAS. Across all three YOLO techniques, the inclusion of dehazing and SAOO-PAS provides better improvement in mAP@0.5 compared to the baseline techniques without dehazing and SAOO-PAS. Thus, the results offer strong proof that the designed image dehazing and SAOO-PAS are highly generalizable and effective across YOLO variants.

### 6.21 Computational complexity analysis of SAOO-PAS

Table 22 provides the computational complexity validation of the designed SAOO-PAS in terms of the total number of fitness evaluations. The suggested SAOO-PAS performs N×T=500 fitness validations to optimize the hyperparameters of the YOLOv9 technique. The designed SAOO-PAS algorithm’s computational complexity is controlled by the training cost of the technique, $O(N\times T\times C_{\text{train}})$ with early convergence happening after 34 iterations. Here, the number of population and the total number of iterations are specified as *N* and *T*. The training cost is specified as $C_{\text{train}}$ . Although the training time increases by nearly 34%, this overhead is reasonable for the large-scale data sources due to the offline execution of the designed SAOO-PAS during training time. Also, the optimized technique does not demand continuous validations at inference time. In addition, the designed SAOO-PAS only performs the hyperparameter optimization. So, it does not introduce any extra parameters or memory limitations at inference time. Hence, it is ensured that the designed SAOO-PAS is more efficient.

**Table 22:** Computational complexity validation of the designed SAOO-PAS in terms of the total number of fitness evaluations.

Metric	Value	Explanation
Population Size (*N*)	10	Number of candidate solutions
Iterations (*T*)	50	Optimization cycles
Total Fitness Evaluations	500	N×T
Complexity Order	$O(N\times T\times C_{\text{train}})$	Dominated by the model training cost
Convergence Iteration	34	Early stabilization
Average Training Time (Baseline)	232 min	Standard YOLOv9
Training Time with SAOO-PAS	311 min	+34% overhead
Inference Parameters Added	0	No architectural modification
Extra Inference Memory	0 MB	Hyperparameters only

### 6.22 Detection performance of proposed model across distinct environmental conditions

**Figure 15:**
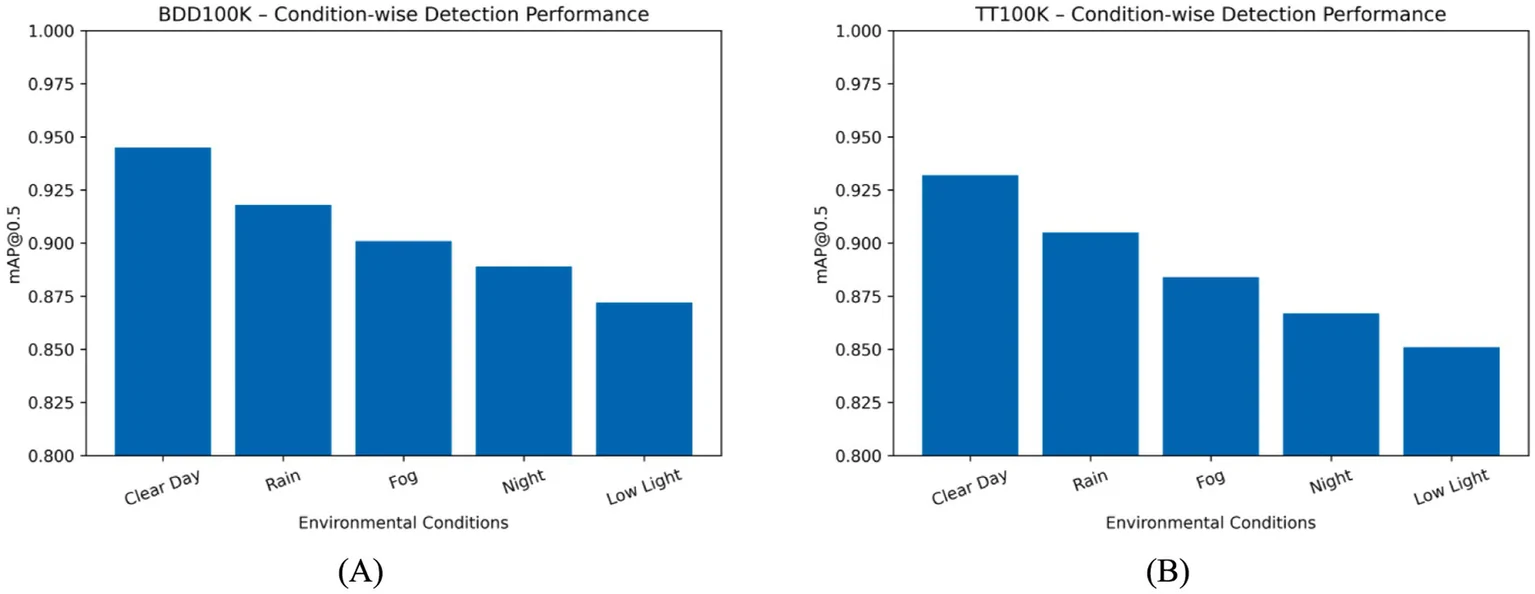
Detection performance of proposed model across distinct environmental conditions in terms of **(A)** BDD100k and **(B)** TT100k.

The suggested MAFF-FPN-AYv9 technique is analyzed in Figure 15 under distinct environmental conditions for two datasets. In this analysis, the environmental conditions, such as a clear day, rain, low light, fog, and night, are considered. The clear day conditions achieve the highest mAP@0.5. It specifies the optimal visibility of the model. The rainy conditions slightly minimize the performance because of the lower contrast. The foggy images processed via the SPP-CAE-based dehazing process provide better detection accuracy. On the other hand, the night and low-light conditions give some low scores. However, the model still identifies most of the traffic signs with the support of the MAFF process. Hence, it is confirmed that the designed MAFF-FPN-AYv9 provides robust detection across distinct environmental conditions.

### 6.23 Dataset splitting and IoU computation during optimization

Originally, the dataset is partitioned into 75% training and 25% testing without explaining the validation set. It may lead to ambiguity in the optimization task. Therefore, in the revised methodology, the dataset partitioning is explained as follows: 60% for training, 25% for testing, and 15% for validation. During training, the parameters of the technique are learned utilizing the training set. The validation set is only utilized for hyperparameter optimization utilizing the SAOO-PAS algorithm. In this phase, the IoU-aided fitness function is estimated. In the final performance reporting, the validation set is not involved. The test set is used as independent data and is employed only once after the optimization task to explain the suggested method’s final performance. This separation guarantees a leakage-free validation and ensures unbiased performance validation. Table 23 gives the detection results of the suggested SAOO-PAS-MAFF-FPN-AYv9 based on the datasets.

**Table 23:** Detection results of the suggested SAOO-PAS-MAFF-FPN-AYv9 based on datasets.

Dataset	Model	IoU	Precision	Recall	mAP
BDD100K	YOLOv9	0.89	0.92	0.90	0.91
BDD100K	SAOO-PAS-MAFF-FPN-AYv9(Proposed)	0.94	0.96	0.95	0.96
TT100K	YOLOv9	0.88	0.91	0.89	0.90
TT100K	SAOO-PAS-MAFF-FPN-AYv9(Proposed)	0.95	0.97	0.96	0.97

### 6.24 Cross-dataset generalization analysis

**Figure 16:**
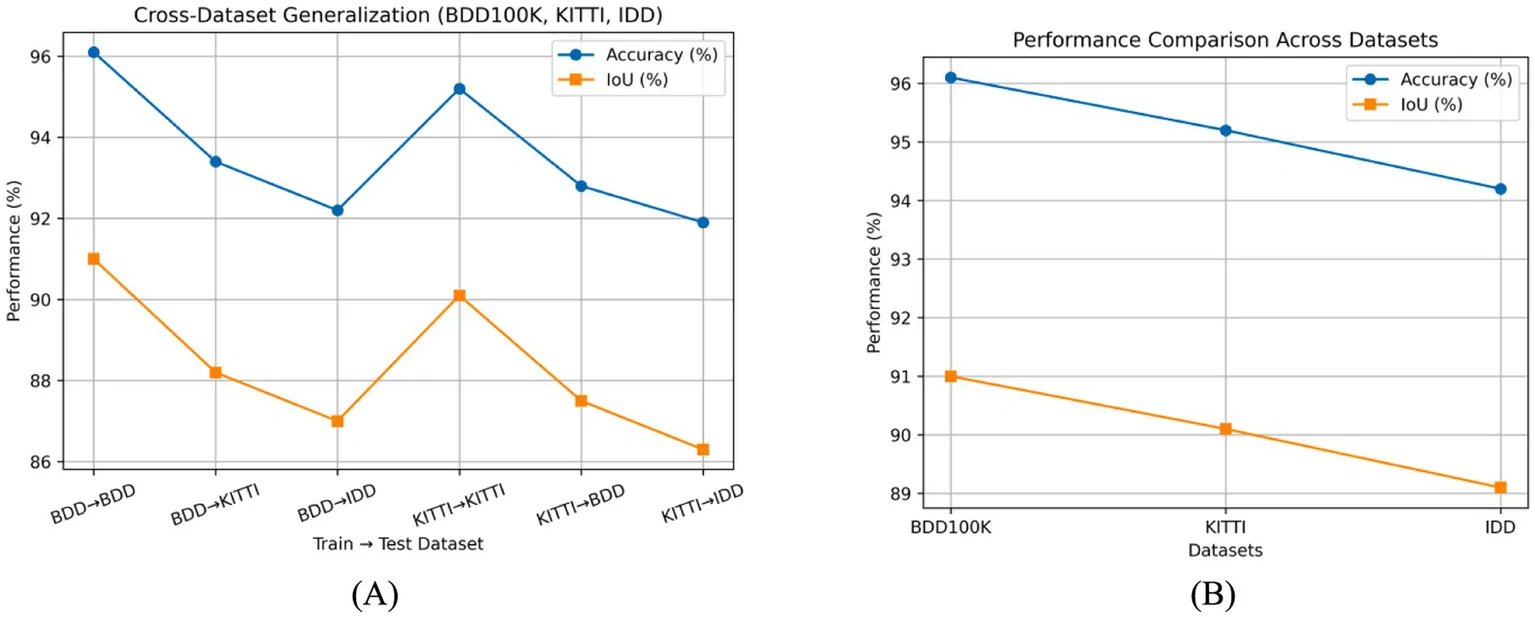
Cross-dataset generalization analysis of the proposed model in terms of **(A)** Train➔Test dataset and **(B)** datasets.

Figure 16 displays the cross-dataset generalization analysis of the suggested model. Here, to further validate the generalization capacity of the designed mechanism, an additional unseen data source, IDD, is utilized for testing without retraining. The link https://idd.insaan.iiit.ac.in/dataset/details/ is supported for accessing this dataset. This data source includes the data gathered from cameras (images), LiDAR sensors (3D point clouds), and GPS/IMU (vehicle motion). To validate the generalization capacity of the designed method under distinct scenarios, the cross-dataset validations are performed across BDD100K, KITTI, and IDD data sources. The performance changes across data sources indicate that the designed approach maintains strong generalization capability, though small performance degradation is monitored when transferring among data sources with distinct environmental conditions. Despite these changes, the designed method maintains high IoU and accuracy rates across all transfer settings. It illustrates strong robustness to domain change. Thus, the results guaranteed that the designed mechanism provides strong cross-domain generalization capacity.

### 6.25 Robustness analysis

**Figure 17:**
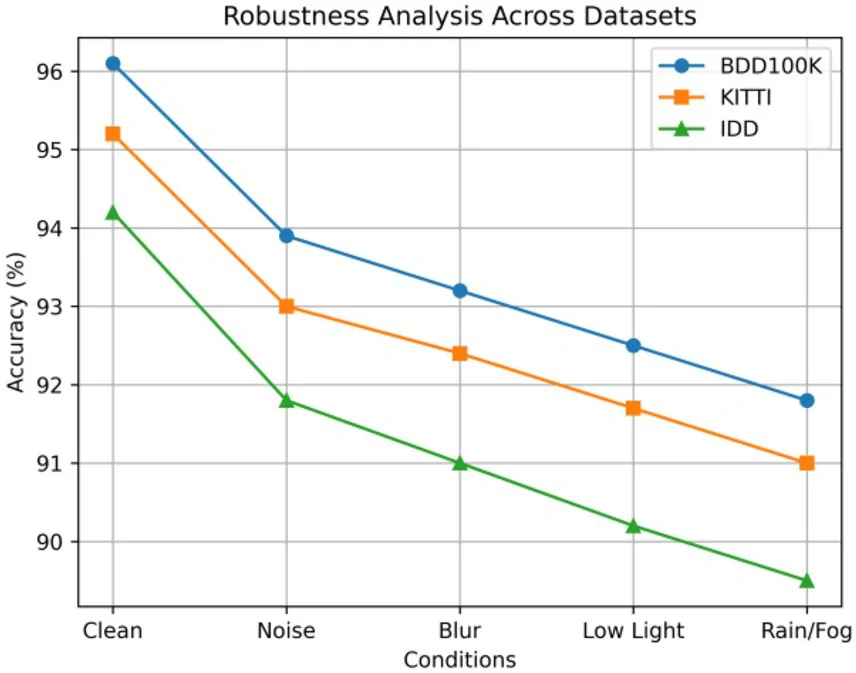
Robustness analysis of the proposed model by considering various environmental conditions.

Figure 17 presents the robustness validation of the designed method. The robustness validation estimates how well the designed method maintains the detection accuracy under critical real-world conditions, such as noise, blur, rain/fog, and low-light environments. As displayed in Figure 17, high performance is achieved under clean conditions, and the performance declines when the distortions increase. Nevertheless, the reduction in accuracy remains moderate across all the data sources. It represents the robustness of the suggested approach. For example, on the BDD100K dataset, the accuracy reduces from 96.1% in the clean scenario to 91.8% under rain/fog. This resilience is attributed to the combination of the SPP-CAE dehazing model, which improves the image quality under low visibility. Also, the MAFF-FPN model highly extracts the multi-scale features even in the degraded inputs. Hence, it is guaranteed that the recommended approach maintains a reliable performance across distinct environmental variations.

### 6.26 Error analysis

**Figure 18:**
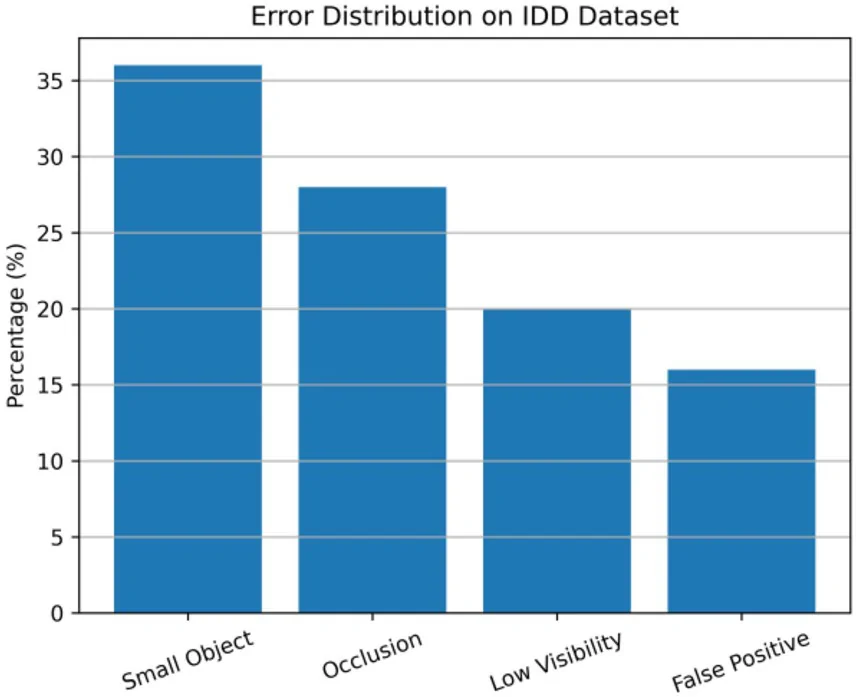
Error analysis of the proposed model.

Figure 18 provides the error analysis of the suggested model. The error validation on the IDD data source gives details on the main detection failure sources for the suggested approach. As displayed in Figure 18, most of the error arises from small object detection. It specifies that, through the utilization of multi-scale feature fusion, the traffic signs with constant pixel representation remain complex. The next factor is occlusion, where the partial obstruction of traffic signs by other objects minimizes the capability of the technique to precisely localize them. The low visibility conditions, such as poor illumination, contribute to 20% of the errors. It elucidates that though the dehazing block improves the quality of an image, some degradation still impacts the feature extraction process. Lastly, the false positives elucidate that the technique maintains better precision with a small number of incorrect detections. Thus, the results elucidate that though the designed approach is robust, further enhancements are required in managing the small-scale objects and occluded scenarios to improve the reliability of the detection process.

### 6.27 State-of-the-art analysis

Table 24 presents the state-of-the-art validation of the recommended traffic sign detection mechanism. This investigation considers various transformer-based baselines. By considering distinct performance measures, the effectiveness of the implemented SAOO-PAS-MAFF-FPN-AYv9 technique is analyzed. From the table results, it is displayed that the designed SAOO-PAS-MAFF-FPN-AYv9’s dice coefficient is 0.955, which is lower than that of the conventional transformer-based methods. In addition, the designed SAOO-PAS-MAFF-FPN-AYv9’s accuracy is showcased as 0.95. From the experimental outcomes, it is ensured that the implemented SAOO-PAS-MAFF-FPN-AYv9 provides more satisfactory outcomes than the previous methods in the traffic sign identification mechanism.

**Table 24:** State-of-the-art analysis.

BDD100K					
Terms	Efficient vision transformer YOLOv5 (Zeng et al., 2024)	Multiscale transformer (Chen et al., 2024)	DETR (Zhang L. et al., 2025)	Transformer-enhanced YOLO architecture (Farhani et al., 2026)	SAOO-PAS-MAFF-FPN-AYv9 (Proposed)
Dice Coefficient	0.940	0.933	0.924	0.940	0.955
Jaccard	0.888	0.874	0.859	0.888	0.914
Accuracy	0.939	0.929	0.923	0.938	0.952
Sensitivity	0.943	0.941	0.925	0.934	0.953
Specificity	0.934	0.915	0.921	0.942	0.951
Precision	0.938	0.925	0.923	0.947	0.957
FPR	0.066	0.085	0.079	0.058	0.049
FNR	0.057	0.059	0.075	0.066	0.047
NPV	0.934	0.915	0.921	0.942	0.951
FDR	0.062	0.075	0.077	0.053	0.043
F1-Score	0.940	0.933	0.924	0.940	0.955
MCC	0.878	0.857	0.846	0.876	0.904

TT100K					
Terms	Efficient vision transformer YOLOv5 (Zeng et al., 2024)	Multiscale transformer (Chen et al., 2024)	DETR (Zhang L. et al., 2025)	Transformer-enhanced YOLO architecture (Farhani et al., 2026)	SAOO-PAS-MAFF-FPN AYv9 (Proposed)
Dice Coefficient	0.936	0.919	0.940	0.932	0.944
Jaccard	0.880	0.850	0.887	0.872	0.894
Accuracy	0.938	0.918	0.941	0.932	0.942
Sensitivity	0.948	0.916	0.943	0.935	0.945
Specificity	0.929	0.920	0.938	0.929	0.938
Precision	0.925	0.922	0.937	0.929	0.943
FPR	0.071	0.080	0.062	0.071	0.062
FNR	0.052	0.084	0.057	0.065	0.055
NPV	0.929	0.920	0.938	0.929	0.938
FDR	0.075	0.078	0.063	0.071	0.057
F1-Score	0.936	0.919	0.940	0.932	0.944
MCC	0.876	0.836	0.881	0.864	0.884

### 6.28 Balance between accuracy gains and computational cost

Though the suggested SAOO-PAS-MAFF-FPN AYv9 model attains better traffic sign identification accuracy, the inclusion of the SPP-CAE image dehazing block, MAFF-FPN, and SAOO-PAS optimization enhances the computational burden contrasted to the existing YOLOv9 model. This additional computational overhead is mainly related to the attention-based feature extraction phase and image enhancement. However, these methods highly enhance the detection robustness and feature representation under adverse weather conditions, small-object scenarios, and complex backgrounds. It leads to higher identification accuracy. Hence, the suggested model represents a practical trade-off between detection performance and computational cost. It makes the model highly applicable for deployment in autonomous driving environments.

## 7 Conclusion

An enhanced traffic sign detection model was developed in this work, which has become an intelligent tool in the area of ITS and autonomous vehicles. The required images were collected and used by the SPP-CAE for the image dehazing process. This model was effective in eliminating the unwanted visibility, which was caused by the inclement weather, thus allowing the proposed system to produce accurate detection results. Then, the dehazed images were provided to the MAFF-FPN-AYv9 for the traffic sign detection purpose. The performance of this model was optimized by the developed SAOO-PAS. The developed traffic sign detection model was evaluated against several state-of-the-art networks, such as YOLOv3, MMW-YOLOv5, YOLOv7, and YOLOv9, in terms of accuracy. Here, the developed SAOO-PAS-MAFF-FPN-AYv9 outperformed the traditional models with 96% accuracy, while the YOLOv3 recorded an accuracy of 84%, MMW-YOLOv5 was 88%, YOLOv7 was 84%, and YOLOv9 was 93%. Thus, the results proved that the developed model was more effective in detecting traffic signs when compared with other models, since it used the power of an enhanced optimization algorithm. This model was a highly helpful tool for autonomous vehicle systems, offering safer and more reliable traffic sign detection. Although the developed model provides high performance, it has some limitations. The developed model requires more hardware, which limits its application in resource-constrained environments. Then, the model’s performance is sometimes mostly based on the quality as well as the diversity of the data used for training the model. Thus, the future work will focus on optimizing and deploying the model to adapt to edge computing devices, which will allow for real-time applications even with a wide variety of vehicles, including those with limited processing power. Finally, to provide accurate and robust detection, the developed model will be incorporated with the sensor data, which includes LiDAR and radar. The suggested model has specific real-time applications, such as smart city management, connected vehicle infrastructure, roadside traffic surveillance, smart traffic monitoring, ITS, Advanced Driver Assistance Systems (ADAS), and autonomous vehicles. In these applications, the designed model gives timely and reliable traffic sign identification for enhancing driving efficiency and road safety.

### 7.1 Limitations and future work

Though the implemented model has been highly validated on the large-scale public data sources, the research on real-time camera or CCTV video streams was not performed in the suggested work. Certain real-time data sources may include additional problems, such as dynamic frame rates, compression artifacts, camera vibration, varying traffic flow, and continuous video processing. Hence, the future work will be performed by estimating the designed MAFF-FPN-AYv9 model employing real-time traffic camera and CCTV footage to further validate its practical deployment in the ITS and autonomous driving applications. In addition, the designed model poses additional computational overhead because of the optimization process and image dehazing. It may constrain the deployment of the suggested model on resource-constrained embedded platforms. To resolve this concern, the future work will be focused on designing lightweight versions of the designed approach for edge computing systems.

## Data Availability

The original contributions presented in the study are included in the article/supplementary material, further inquiries can be directed to the corresponding author/s.
